# Type 2 diabetes is associated with increased circulating levels of 3-hydroxydecanoate activating GPR84 and neutrophil migration

**DOI:** 10.1016/j.isci.2022.105683

**Published:** 2022-11-26

**Authors:** Randi Bonke Mikkelsen, Tulika Arora, Kajetan Trošt, Oksana Dmytriyeva, Sune Kjærsgaard Jensen, Abraham Stijn Meijnikman, Louise Elisabeth Olofsson, Dimitra Lappa, Ömrüm Aydin, Jens Nielsen, Victor Gerdes, Thomas Moritz, Arnold van de Laar, Maurits de Brauw, Max Nieuwdorp, Siv Annegrethe Hjorth, Thue Walter Schwartz, Fredrik Bäckhed

**Affiliations:** 1 Novo Nordisk Foundation Center for Basic Metabolic Research, University of Copenhagen, Copenhagen, Denmark; 2 Department of Internal and Vascular Medicine, Amsterdam University Medical Centre, Amsterdam, the Netherlands; 3 Department of Molecular and Clinical Medicine/Wallenberg Laboratory, Sahlgrenska Academy, University of Gothenburg, Gothenburg, Sweden; 4 Department of Biology and Biological Engineering, Chalmers University of Technology, Gothenburg, Sweden; 5 Department of Surgery, Spaarne Hospital, Hoofddorp, the Netherlands; 6 Region Västra Götaland, Sahlgrenska University Hospital, Department of Clinical Physiology, Gothenburg, Sweden

**Keywords:** Pathophysiology, Immunology, Cell biology

## Abstract

Obesity and diabetes are associated with inflammation and altered plasma levels of several metabolites, which may be involved in disease progression. Some metabolites can activate G protein-coupled receptors (GPCRs) expressed on immune cells where they can modulate metabolic inflammation. Here, we find that 3-hydroxydecanoate is enriched in the circulation of obese individuals with type 2 diabetes (T2D) compared with nondiabetic controls. Administration of 3-hydroxydecanoate to mice promotes immune cell recruitment to adipose tissue, which was associated with adipose inflammation and increased fasting insulin levels. Furthermore, we demonstrate that 3-hydroxydecanoate stimulates migration of primary human and mouse neutrophils, but not monocytes, through GPR84 and Gα_i_ signaling *in vitro*. Our findings indicate that 3-hydroxydecanoate is a T2D-associated metabolite that increases inflammatory responses and may contribute to the chronic inflammation observed in diabetes.

## Highlights

• 3-hydroxydecanoate is increased in T2D compared with individuals without T2D
• 3-hydroxydecanoate increases WAT inflammation and neutrophil recruitment
• 3-hydroxydecanoate is a GPR84 agonist
• 3-hydroxydecanoate mediates neutrophil migration through GPR84 and Gα_i_ signaling

## Graphical abstract

**Figure:**
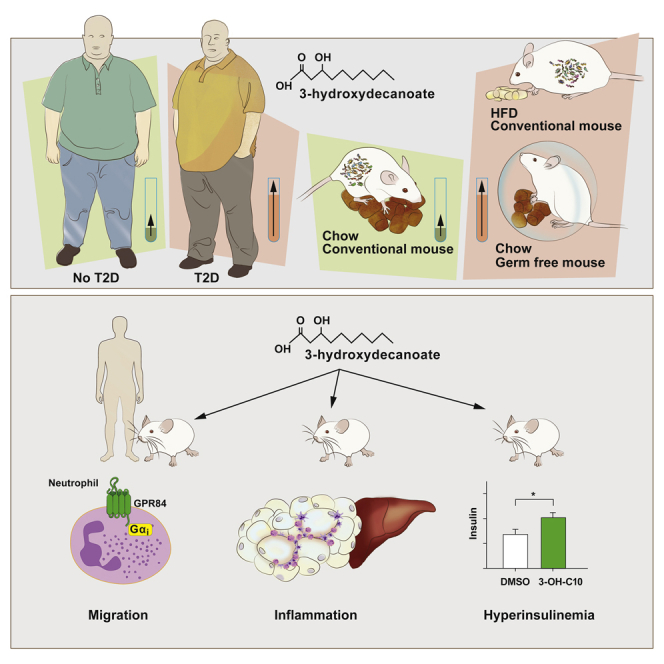

## Introduction

Obesity and diabetes are global epidemics with more than 1.9 billion people being obese or overweight^1^ and 463 million adults having diabetes.^2^ Obesity is the most common cause of several metabolic defects and is associated with complications such as type 2 diabetes (T2D) and cardiovascular disease.^3^^,^^4^ Yet, a deeper understanding of the molecular mechanisms behind these defects is still lacking.

Diabetes and other metabolic diseases are associated with dysmetabolism and changed levels of several plasma metabolites including branched-chain amino acids (e.g. leucine, isoleucine, and valine^5^^,^^6^^,^^7^; free fatty acids,^6^^,^^7^^,^^8^ bile acids, and microbial metabolites such as short-chain fatty acids (SCFAs), and small intermediary metabolites like succinate or lactate.^6^^,^^9^^,^^10^^,^^11^

Some disease-regulated metabolites elicit effects through G protein-coupled receptors (GPCRs).^12^^,^^13^ GPCRs are expressed in e.g. immune cells, adipocytes, and endocrine cells and are involved in functions such as lipolysis, gut hormone secretion, insulin secretion, and chemotaxis.^9^^,^^10^ Plasma levels of the SCFA acetate are increased in diabetes and inhibit insulin secretion through the free fatty acid receptor 2 (FFA2/GPR43) and FFA3 (GPR41).^9^^,^^14^ In addition, lactate acting through GPR81 (HCA1),^15^ succinate acting through GPR91, 3-hydroxybutyrate acting through GPR109A (HCA2), and 3-hydroxyoctanoate acting through GPR109B (HCA3) inhibited lipolysis in adipocytes.^9^ However, GPR109B is not expressed in mice.^16^ Some metabolites and GPCRs have also demonstrated pro- or anti-inflammatory effects.^9^ For example, butyrate increased *Il10* expression in murine macrophages and modulated inflammation in a mouse model in a GPR109A-dependent manner, suggesting anti-inflammatory functions.^17^ In addition, the SCFAs butyrate, acetate, and propionate increased the number of regulatory T cell in the colon of mice. Furthermore, the suppressive effect of propionate on effector T cells was dependent on FFA2 signaling.^18^ Feeding mice omega-3-free fatty acids resulted in increased numbers of anti-inflammatory M2 macrophages in adipose tissue, mediated through GPR120 (FFA4).^19^^,^^20^ Succinate, on the other hand, worsened disease in a murine colitis model,^21^ while GPR91 knockout (KO) macrophages demonstrated reduced release of tumor necrosis factor alpha (TNF-α) and interleukin 1β (IL-1β).^22^

GPR84 is a medium-chain fatty acid (MCFA) receptor, with decanoate (C10) being the suggested endogenous agonist, but with MCFAs with shorter and longer acyl chains being less potent agonists. GPR84 is highly expressed in immune-related tissues and cells, e.g. monocytes, neutrophils, and macrophages, particularly those of the M1 phenotype.^23^^,^^24^^,^^25^^,^^26^ Gene expression of *Gpr84* is increased in macrophages, adipose tissue, kidney, and intestines in mice after LPS injection; in kidneys of diabetic NOD mice^27^; in kidneys of mice with acute and chronic kidney injuries^26^; and in peripheral blood mononuclear cells from patients with systemic lupus erythematosus and the complication lupus nephritis.^28^ Based on its expression pattern and cellular effects, GPR84 is considered a predominantly pro-inflammatory receptor involved in inflammatory gene expression, cytokine release, and neutrophil migration.^23^^,^^26^^,^^29^^,^^30^^,^^31^ GPR84 is reported to recruit β-arrestin^32^ and to signal through Gα_i_ and Gα_12/13_, but not through Gα_q_ or Gα_s_.^23^^,^^29^^,^^33^ Importantly, this dual signaling through Gα_i_ and Gα_12/13_ is characteristic for chemotactic receptors,^33^ and the GPR84 sequence in fact resembles chemotactic receptors.^24^ In addition, GPR84 mediates laurate (C12)-stimulated insulin secretion in murine pancreatic islets^34^ and regulates insulin resistance, glycemic control,^35^ and lipid metabolism.^36^ Furthermore, a GPR84 antagonist alleviated disease in a murine dextran sulfate sodium-induced colitis model^37^ and reduced inflammation in acute liver injury.^38^ Overall, these findings indicate a role of GPR84 in metabolism and inflammation *in vivo*. As such, multiple studies indicate that (disease-regulated) metabolites could be involved in physiological or pathological mechanisms through GPCR signaling.

In obesity, the adipose tissue secretes cytokines and chemokines such as TNF-α and monocyte chemoattractant protein 1 (MCP-1),^39^^,^^40^^,^^41^^,^^42^^,^^43^ resulting in infiltration of neutrophils,^44^ monocytes, and differentiation to predominantly M1 macrophages.^40^^,^^43^^,^^45^^,^^46^^,^^47^^,^^48^ The increased inflammation resulting from recruitment of immune cells contributes to the development of insulin resistance, e.g. by decreasing expression of *Glut4* in adipocytes and attenuating insulin signaling.^3^^,^^4^^,^^39^^,^^49^^,^^50^ Conversely, genetic deletion of MCP-1,^41^ depletion of CD11c^+^ macrophages,^51^ or genetic deletion of the neutrophil protease elastase^44^ reduced adipose tissue neutrophil and macrophage infiltration, tissue inflammation, and protected from insulin resistance. Similarly, neutrophils are also recruited to the liver in response to high-fat diet (HFD) in mice and can induce cellular insulin resistance in hepatocytes.^44^ Since multiple metabolite-sensing GPCRs have demonstrated inflammatory effects, it is possible that they modulate these inflammatory responses leading to insulin resistance and ultimately T2D.

Since cardiometabolic disease involves intricate signaling between different tissues in the body, we designed the BARIA study to investigate how obese patients with or without T2D differed in metabolic and anthropometric characteristics, gut microbiota, circulating metabolites, and tissue responses in patients undergoing bariatric surgery.^52^ We have identified metabolites associated with T2D using metabolomics of peripheral plasma before and after a mixed meal test (MMT). We identified the MCFA 3-hydroxydecanoate to be positively correlated with fasting glucose, HbA1c, and HOMA-IR.^53^ The metabolite structurally resembles decanoate and 3-hydroxyoctanoate. These lipids are agonists for GPR84^9^^,^^23^^,^^29^ and GPR109B,^9^ respectively, and 3-hydroxydecanoate has indeed been suggested to be an agonist of these two GPCRs.^29^^,^^54^ Here, we addressed whether the T2D-associated metabolite 3-hydroxydecanoate can contribute to altered metabolism and examined if it signals through GPR84.

## Results

### 3-Hydroxydecanoate is enriched in obese patients with T2D

**Figure 1:**
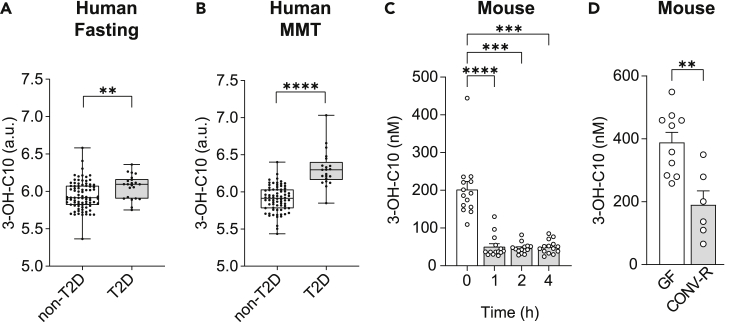
3-hydroxydecanoate is enriched in obese patients with T2D and fasting induced in mice (A and B) Peripheral plasma levels of 3-hydroxydecanoate (3-OH-C10) in obese individuals with or without T2D at A) fasting and B) after a 2 h mixed meal test (MMT). The data are presented as boxplots where the box shows the 25^th^, median and 75^th^ percentiles. The whiskers show minimum and maximum. (C) Plasma levels of 3-hydroxydecanoate in eye blood from CONV-R (N = 14) mice. The mice were fasted for 16 h followed by refeeding and sampling for 4 h. (D) Plasma levels of 3-hydroxydecanoate in eye blood from GF and CONV-R mice after fasting for 4 h (N = 6–10 mice/group). Data are shown as mean ± SEM (C and D). ∗∗p < 0.01, ∗∗∗p < 0.001, ∗∗∗∗p < 0.0001. p values were determined by two-tailed Mann-Whitney test (A, B, and D) or Friedman’s test with Dunn’s post-hoc analysis (C). See also Figure S1.

T2D has previously been associated with altered plasma levels of various metabolites,^5^^,^^7^^,^^8^ and untargeted metabolomics in plasma from 106 obese individuals identified plasma levels of 3-hydroxydecanoate to be associated with fasting glucose, HbA1c, and HOMA-IR.^53^ Furthermore, 3-hydroxydecanoate (3-OH-C10) was increased in obese individuals with T2D compared with obese individuals without T2D (Table 1) in fasting plasma samples (Figure 1A) and following a mixed meal test (MMT) (Figure 1B), indicating differential metabolism or absorption of 3-hydroxydecanoate in T2D.

**Table 1:** Baseline characteristics of the BARIA cohort.

	NGT and pre-DM (N = 84)	T2D (N = 22)
Demographic		
Age (*years*)	45.4 ± 10.0	47.1 ± 10.1
Female - no. (%)	68 (81.0)	16 (72.7)

**Anthropometric**		

BMI (*kg/m*^*2*^)	40.0 (37.4–41.2)	38.7 (35.9–42.7)
Weight (*kg*)	117.0 (106.0–126.3)	118.5 (106.9–126.6)

**Laboratory results**		
Fasting glucose (*mmol/L*)	5.6 (5.2–6.0)	7.7 (6.8–8.9)∗
HbA1c (*mmol/mol*)	37 (34.0–40.0)	52.0 (46.0–60.0)∗
Fasting insulin (*pmol/L*)	79 (55–112)	119.0 (64.3–212.3)∗
Total cholesterol (*mmol/L*)	5.0 ± 1.1	4.3 ± 1.0∗∗
HDL-cholesterol (*mmol/L*)	1.2 (1.0–1.4)	1.0 (0.9–1.2)
LDL-cholesterol (*mmol/L*)	3.3 ± 1.0	2.7 ± 0.8∗
Triglycerides (*mmol/L*)	1.3 (1.0–1.8)	1.6 (1.3–1.8)
CRP (*mg/L*)	5.0 (2.9–7.9	5.3 (3.2–9.4

Groups are divided into individuals with a normal glucose tolerance (NGT) or pre-diabetes (pre-DM) vs. type 2 diabetes (T2D) based on the American Diabetes Association criteria (ADA). Results are expressed as means ± SD For categorical variables, number and percentages are presented. Non-normally distributed variables are presented as median with interquartile range. For comparison between groups, Fisher's Exact test was used for dichotomous variables and Student’s t-test or Wilcoxon rank-sum test were used as appropriate for continuous variables.

∗p < 0.05, ∗∗p < 0.01. BMI, body mass index; CRP, C-reactive protein; HbA1c, Hemoglobin A1c; HDL, high-density lipoprotein; LDL, low-density lipoprotein.

### 3-Hydroxydecanoate is fasting induced and enriched in GF mice

To further investigate the regulation of 3-hydroxydecanoate levels in fasting and fed states, we fasted mice overnight and refed them chow diet for 4 h. Importantly, 3-hydroxydecanoate was not present in the chow diet (Figure S1A), indicating that any regulation of 3-hydroxydecanoate plasma levels *in vivo* would be due to endogenous metabolism. The plasma levels of 3-hydroxydecanoate were significantly reduced following the refeeding (Figure 1C), which could suggest that 3-hydroxydecanoate is released during fasting-induced β-oxidation, as previously described.^55^ In agreement with increased β-oxidation, we observed that 3-hydroxydecanate was present in the liver, but not in epididymal white adipose tissue (eWAT) (Figure S1A). Mice that were fed HFD for two weeks had significantly higher plasma levels of 3-hydroxydecanoate than mice fed chow (Figure S1B), although the HFD did not contain 3-hydroxydecanoate (Figure S1A).

Since the gut microbiota interacts with the diet and produces bioactive metabolites regulating host metabolism,^11^ we investigated if 3-hydroxydecanoate was regulated by the gut microbiota. 3-hydroxydecanoate levels were higher in germ-free (GF) compared with conventionally raised mice (CONV-R) (Figure 1D), which suggests that 3-hydroxydecanoate is not produced by the microbiota, but the gut microbiota may still regulate its levels.

### 3-Hydroxydecanoate increases fasting insulin in mice

**Figure 2:**
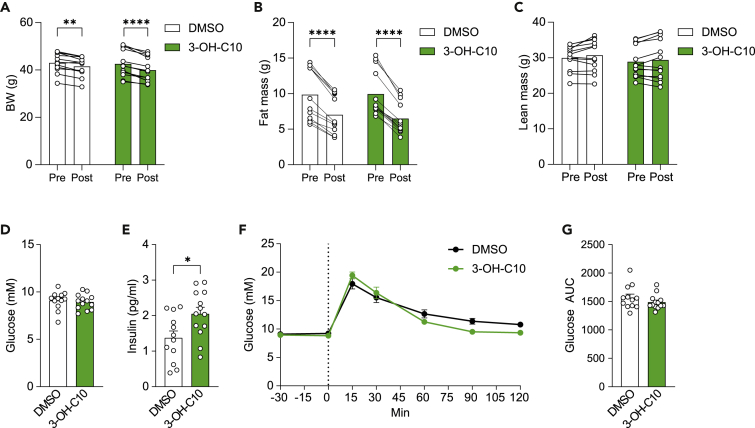
3-Hydroxydecanoate increases fasting insulin in mice (A–C) (A) Body weight (BW), (B) fat mass, and (C) lean mass in mice before (“Pre”) and after (“Post”) i.p. dosing with 3-hydroxydecanoate (3-OH-C10, 25 mg/kg) or vehicle (DMSO) for seven days. (D and E) Fasting (4 h) (D) blood glucose and (E) serum insulin in mice after i.p. dosing with 3-hydroxydecanoate (25 mg/kg) or vehicle (DMSO) for seven days. The measurements were performed 30 min before dosing glucose for the ipGTT. (F and G) (F) Glucose levels and (G) AUC during an intraperitoneal glucose tolerance test (ipGTT) in mice dosed i.p. with 3-hydroxydecanoate or vehicle for seven days. N = 12–13 mice/group. Data are shown as mean ± SEM ∗p < 0.05, ∗∗p < 0.01, ∗∗∗∗p < 0.0001. p values were determined by two-way ANOVA followed by Sidak’s multiple comparison test (A–C, F–H) or Mann-Whitney test (D, E, and G).

Since 3-hydroxydecanoate levels were elevated in T2D compared with obese nondiabetic controls (Figures 1A and 1B), we next examined if 3-hydroxydecanoate could affect glucose metabolism in mice. Mice were treated with daily intraperitoneal (i.p.) injections of 3-hydroxydecanoate for seven days. Both the vehicle- and 3-hydroxydecanoate-treated groups decreased slightly in body weight (Figure 2A), which was mainly attributed to decreased fat mass, suggesting this effect was due to the procedure (Figures 2B and 2C). Fasting glucose was not different between the groups (Figure 2D), while fasting insulin was significantly increased in the 3-hydroxydecanoate-treated group (Figure 2E). To investigate if 3-hydroxydecanate affected glucose tolerance, we next performed intraperitoneal glucose tolerance test in mice treated with vehicle or 3-hydroxydecanoate for seven days, but did not observe any differences in glucose tolerance (Figures 2F and 2G). Thus, this suggests that 3-hydroxydecanoate may affect early stages of impaired glucose metabolism that has not yet developed into glucose intolerance.

### 3-Hydroxydecanoate increases adipose tissue inflammation in mice

**Figure 3:**
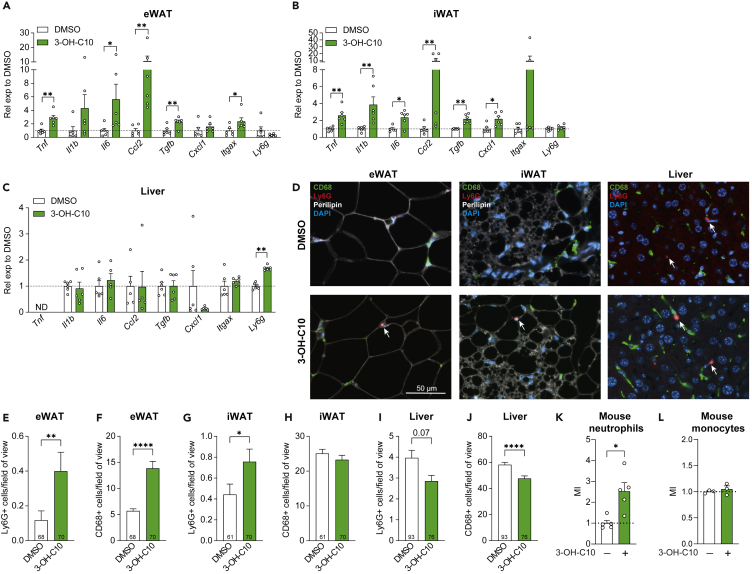
3-Hydroxydecanoate increases adipose tissue inflammation in mice (A–C) Expression of inflammatory genes in CONV-R mice after i.p. dosing with 3-hydroxydecanoate (3-OH-C10, 25 mg/kg) or vehicle (DMSO) for seven days. Gene expression was analyzed by qRT-PCR in (A) eWAT, (B) iWAT, and (C) the liver one day after the last dosing (N = 6 mice/group). (D) Representative images of Ly6G^+^ neutrophils (red; white arrows) and CD68^+^ macrophages (green) in eWAT, iWAT, and liver after dosing DMSO vehicle or 3-hydroxydecanoate for seven days. Cell nuclei were stained with DAPI (blue), and perilipin is white. Scale bar represents 50 μm. Images were taken at 20× magnification. (E–J) Quantification of Ly6G^+^ and CD68^+^-positive cells in (E and F) eWAT, (G and H) iWAT, and (I and J) liver. The data are presented as the average number of positive cells per field of view from N = 6 mice/group. The number of counted sections is indicated within each bar. (K) Migration of murine bone-marrow-derived neutrophils toward 100 μM 3-hydroxydecanoate or DMSO vehicle. The migration index (MI) represents the migration relative to the vehicle control (N = 5). (L) Migration of murine bone-marrow-derived monocytes toward 100 μM 3-hydroxydecanoate or DMSO vehicle (N = 3). Data are shown as mean ± SEM, ND = Not detected. ∗p < 0.05, ∗∗p < 0.01, ∗∗∗∗p < 0.0001. p values were determined by two-tailed Mann-Whitney test. See also Figure S2.

Obesity and diabetes are associated with low-grade inflammation in white adipose tissue (WAT) and several lipid mediators, including 3-hydroxydecanoate, can modulate inflammation.^56^^,^^57^ Therefore, we assessed whether i.p. administration of 3-hydroxydecanoate affected inflammatory gene expression in eWAT, inguinal WAT (iWAT), and the liver (Figures 3A–3C). The expression of *Tnf*, *Ccl2*, and *Il6* was significantly increased in both fat depots, and *Il1b* expression was significantly increased in the iWAT, suggesting that 3-hydroxydecanoate increases inflammation in adipose tissues. *Cxcl1*, encoding the potent neutrophil chemokine CXCL1 (or KC/GRO), which is a murine homolog of the human chemokines CXCL1 and CXCL8/IL-8,^58^^,^^59^^,^^60^ was also significantly increased in iWAT (Figure 3B). Interestingly, *Itgax*, which is commonly used to identify macrophages and dendritic cells,^40^^,^^47^ was significantly increased in eWAT (Figure 3A), while the murine neutrophil marker *Ly6g*^61^ was significantly increased only in the liver (Figure 3C). The plasma levels of IL-1β, IL-6, TNF-α, and KC/GRO were not different between the groups (Figures S2A–S2D), indicating that 3-hydroxydecanoate did not induce systemic inflammation, while leading to inflammation in metabolic tissues.

We next investigated if 3-hydroxydecanoate increased accumulation of immune cells in the tissues by staining for the macrophage marker CD68 and the neutrophil marker Ly6G (Figure 3D). The numbers of both Ly6G^+^ neutrophils and CD68^+^ macrophages were increased in eWAT, consistent with increased gene expression of *Itgax* in eWAT after 3-hydroxydecanoate administration (Figures 3D–3F). Furthermore, 3-hydroxydecanoate dosing resulted in a significant increase in Ly6G^+^ neutrophils in iWAT (Figures 3D and 3G), while there was no difference between the numbers of CD68^+^ macrophages (Figures 3D and 3H). In the liver, we observed a trend toward decreased numbers of liver neutrophils (p = 0.07), despite increased *Ly6g* expression (Figures 3C–3D and 3I); as well as a significant reduction in macrophages after dosing 3-hydroxydecanoate (Figures 3D and 3J). Thus overall, 3-hydroyxdecanoate appears to increase infiltration of neutrophils and macrophages in adipose tissue.

To investigate if 3-hydroxydecanoate directly stimulated migration of the immune cells, we performed Transwell migration experiments with primary murine immune cells and observed that murine bone-marrow-derived neutrophils migrated toward 3-hydroxydecanoate (Figure 3K). In contrast, 3-hydroxydecanoate did not affect monocyte migration (Figure 3L), suggesting that 3-hydroxydecanoate may directly activate neutrophil migration while macrophage recruitment could be indirectly regulated.

### 3-Hydroxydecanoate signals through Gα_i_ and Gα_q_

**Figure 4:**
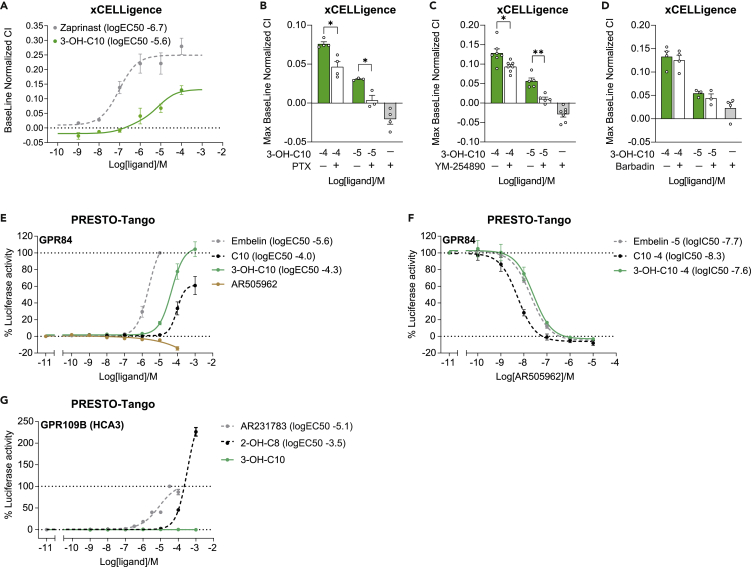
3-Hydroxydecanoate signals through Gα_i_, Gα_q_, and GPR84 (A) Baseline normalized cell index (CI) of cells stimulated with 3-hydroxydecanoate (3-OH-C10) or the positive control compound, zaprinast, in the xCELLigence assay (N = 3). (B–D) Maximum baseline normalized CI of cells stimulated with 3-hydroxydecanoate after pre-incubation with (B) the Gα_i_ inhibitor PTX (200 ng/mL) for 16 h (N = 3–4), (C) the Gα_q_ inhibitor YM-254890 (2 μM) for 15 min (N = 5–7), or (D) the β-arrestin inhibitor barbadin (1 μM) for 15 min (N = 3–4). (E and F) PRESTO-Tango luciferase assay in HTLA cells transfected with human GPR84. (E) Transfected cells were stimulated with 3-hydroxydecanoate, the endogenous GPR84 agonist decanoate (capric acid, C10), the GPR84 reference agonist embelin or the GPR84 antagonist AR505962 (N = 4–5). (F) Transfected cells were pre-incubated with the GPR84 antagonist AR505962, followed by stimulation with sub-maximal concentrations of 3-hydroxydecanoate, decanoate, or embelin (N = 3–4). (G) PRESTO-Tango assay in HTLA cells transfected with human GPR109B (HCA3). The cells were stimulated with 3-hydroxydecanoate, the endogenous GPR109B agonist 2-hydroxyoctanoate (2-OH-C8), or the GPR109B reference agonist AR231783. N = 3. Data are shown as mean ± SEM The logEC50 values were calculated in GraphPad Prism using nonlinear regression.∗p < 0.05, ∗∗p < 0.01. p values were determined by one-way ANOVA followed by Tukey’s multiple comparison test (B–D). See also Figure S3.

Because we found that 3-hydroxydecanoate induced an immunometabolic response in mice, we sought to identify potential receptors for the metabolite in order to further study the mechanisms behind its effects. We first performed the label-free signaling assay xCELLigence, where an electric current is sent through the cell culture plate, and changes in the electrical impedance are measured in real time to record changes in cell adhesion, e.g. due to cell growth, migration, or viability due to altered signaling.^62^^,^^63^ We found that 3-hydroxydecanoate induced a dose-dependent effect on the cell index (CI), indicating that it can activate cellular responses (Figures 4A, S3A, and S3B). Although the closely related fatty acid decanoate (C10) showed minor effect on the CI (Figure S3C), we could not determine a dose-response curve and EC50 value for decanoate, demonstrating how a single hydroxy group can change the signaling effect of a molecule.

Since several metabolites are ligands for GPCRs and 3-hydroxydecanoate has been suggested to activate two GPCRs, GPR84 and GPR109B,^29^^,^^54^ we next investigated if the effect of 3-hydroxydecanoate in the xCELLigence assay was mediated through GPCR signaling. We therefore pre-incubated the cells with inhibitors of various GPCR signaling pathways, as previously reported.^13^^,^^63^^,^^64^ First, we pre-incubated the cells with pertussis toxin (PTX) to inhibit signaling through Gα or M−254890 that inhibits Gα_q_.^65^ Pre-incubation of cells with PTX (Figure 4B) or YM-254890 (Figure 4C) significantly decreased the effect of the highest tested concentration of 3-hydroxydecanoate, and almost completely inhibited the effect of the lowest tested, suggesting that 3-hydroxydecanoate signals through both Gα_i_ and Gα_q_ pathways. Most GPCRs also recruit β-arrestin, which can lead to receptor internalization, signal transduction through ERK1/2, but also signal termination.^66^ Therefore, we pretreated the cells with the β-arrestin/β2-adaptin inhibitor barbadin^67^ to investigate if β-arrestin was involved in the signaling effects of 3-hydroxydecanoate. However, barbadin did not inhibit signaling by 3-hydroxydecanoate (Figure 4D), indicating that the CI changes induced by 3-hydroxydecanoate are not dependent on β-arrestin/β2-adaptin-mediated endocytosis. Taken together, our data support that 3-hydroxydecanoate signals through G protein-dependent pathways.

### 3-Hydroxydecanoate is a GPR84 agonist

We next tested the signaling by 3-hydroxydecanoate in a number of different GPCR-specific signaling assays in order to identify or verify potential GPCR targets for 3-hydroxydecanoate. First, we studied signaling using the β-arrestin-based assay PRESTO-Tango, where each GPCR is engineered to be able to elicit β-arrestin signaling, even if the native GPCR does not.^68^^,^^69^ Since 3-hydroxydecanoate signals through at least two different pathways, Gα_i_ and Gα_q_ (Figures 4B and 4C), the PRESTO-Tango assay allowed us to study receptor signaling in an unbiased manner, i.e. without having to consider which downstream pathway may be initiated by 3-hydroxydecanoate on any given GPCR. Since it has previously been described that 3-hydroxydecanoate could be an agonist for GPR84 and GPR109B,^54^ we used the PRESTO-Tango assay to investigate signaling in an unbiased manner through these two receptors. We found that 3-hydroxydecanoate was a potent agonist for human GPR84 (Figure 4E), yet not as potent as the reference agonist embelin. However, 3-hydroxydecanoate appeared to be a more potent and efficacious agonist for GPR84 than the reported endogenous agonist for GPR84, decanoate (C10)^23^ (Figure 4E). Next, we stimulated GPR84-expressing HTLA cells with a sub-maximal concentration of each GPR84 agonist along with increasing concentrations of the GPR84 antagonist AR505962. Here, 3-hydroxydecanoate signaling was inhibited by AR505962 (Figure 4F), as was the signaling by embelin and decanoate. Of note, AR505962 was more potent in inhibiting decanoate signaling, but this is likely due to the fact that decanoate had reduced capacity to activate luciferase activity at the 100 μM (−4) concentration compared with 3-hydroxydecanoate (Figure 4E). In contrast, 3-hydroxydecanoate did not signal through GPR109B, while the synthetic and endogenous reference agonists AR231783 and 2-hydroxyoctanoate (2-OH-C8) did (Figure 4G).

### 3-Hydroxydecanoate signals through GPR84-Gα_i_

**Figure 5:**
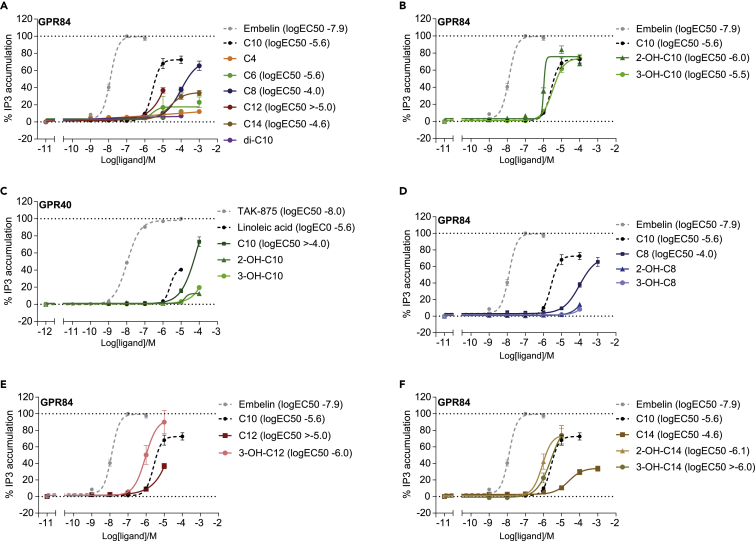
3-Hydroxydecanoate signals through GPR84-Gα_i_ (A) IP3 accumulation assay of COS-7 cells transfected to co-express human GPR84 and the chimeric G protein Gα_Δ6qi4myr_. The cells were stimulated with saturated fatty acids with acyl chains of four to 14 carbons (C4-C14, di-C10) as well as the GPR84 reference agonist embelin. (B) Human GPR84-transfected cells were stimulated with 3-hydroxydecanoate (3-OH-C10), 2-hydroxydecanoate (2-OH-C10), decanoate (C10), and the GPR84 reference agonist embelin (N = 3–5 for MCFAs, N = 6 for embelin). (C) Cells transfected to express human GPR40 were stimulated with 3-hydroxydecanoate, 2-hydroxydecanoate, decanoate and the endogenous and synthetic GPR40 agonists, linoleic acid, and TAK-875^70^ (N = 3–5). (D–F) Human GPR84- and Gα_Δ6qi4myr_-transfected cells were stimulated with (D) octanoate (C8), (E) laurate (C12, dodecanoate), (F) myristate (C14, tetradecanoate), as well as the 2- and 3-hydroxy-derivatives of the respective acyl chain lengths. (A, B, and D–F) (N = 3–5), except for C10 (N = 7) and embelin (N = 8). Data for embelin and non-hydroxylated fatty acids in (A) are replicated in (B and D–F) as relevant, to serve as controls. Data are shown as mean ± SEM The logEC50 values were calculated in GraphPad Prism using nonlinear regression. For unsaturated curves, the potencies are indicated as being above a certain concentration. Absence of a logEC50 value indicates that this could not be calculated. See also Figures S4, S5 and Table S1.

To further explore the signaling induced by 3-hydroxydecanoate on GPR84 or any potential additional GPCRs, we next performed inositol trisphosphate (IP3) accumulation assays in GPR84-transfected cells (Figure 5, Table S1). GPR84 is known as a Gα_i_-coupled receptor^23^^,^^29^^,^^33^ and thus does not itself stimulate IP3 production, which depends on Gα_q_ signaling.^71^ However, by co-transfecting cells with the GPCR of interest and the chimeric G protein Gα_Δ6qi4myr_,^72^ intracellular receptor signaling can be diverted from Gα_i_ to Gα_q_, which allows comparison within the same assay.

Using the IP3 accumulation assay, we first verified previous findings^23^ that decanoate (C10) is a more potent GPR84 agonist than octanoate (C8) and myristate (C14, tetradecanoate) (Figure 5A, Table S1). Laurate (C12, dodecanoate) also appeared to be less potent than decanoate, yet since an accurate EC50 value could not be determined for laurate, this could not be firmly concluded. Butyrate (C4) and hexanoate (C6), being shorter than the expected agonists for GPR84, showed limited if any potency and efficacy through GPR84 (Figure 5A). Similarly, sebacate (decanedioate, di-C10) having 10 carbons like decanoate, but two carboxylic acid groups, did not signal through GPR84 (Figure 5A). Importantly, we next verified that both human (Figure 5B, Table S1) and murine GPR84 (Figure S4A) are indeed receptors for 3-hydroxydecanoate, with logEC50 values of −5.5 and −5.7, respectively. Interestingly, in contrast to the PRESTO-Tango and xCELLigence assays, 3-hydroxydecanoate and decanoate showed almost identical potencies and efficacies on GPR84 in the IP3 assay (Figure 5B, Table S1), suggesting biased signaling of these GPR84 agonists.

Finally, we tested the signaling of 3-hydroxydecanoate on additional metabolite-sensing GPCRs (Table S1). Since the fatty acid receptors GPR40 and GPR120 can bind metabolites structurally similar to 3-hydroxydecanoate,^9^^,^^73^ we next investigated signaling through these. Interestingly, we observed a minor effect by 3-hydroxydecanoate on the Gα_q_-coupled GPR40 (Figure 5C), yet without being able to calculate an EC50 value. However, this could potentially explain the effect of YM-254890 on 3-hydroxydecanoate signaling in the xCELLigence assay (Figure 4C). In contrast, we did not observe any signaling of 3-hydroxydecanoate through the Gα_q_- and Gα_i_-coupled GPR120 (Figure S4B). We also confirmed the PRESTO-Tango data that 3-hydroxydecanoate did not signal through the Gα_i_-coupled GPR109B (Figure S4C, Table S1), nor any other tested metabolite-sensing GPCR using the IP3 accumulation assay (Table S1).

### 3-Hydroxydecanoate does not recruit β-arrestin

Since most GPCRs signal through β-arrestin,^66^ we also screened 3-hydroxydecanoate on 241 GPCRs in the PathHunter GPCR β-Arrestin recruitment assay to search for any potential additional GPCR targets (Figures S5A–S5E). Yet, no signaling was observed by 3-hydroxydecanoate on any GPCRs in this assay, including GPR84 and GPR109B (Figures S5A–S5E), suggesting that 3-hydroxydecanoate does not induce β-arrestin recruitment and may thus be a biased agonist, which is in agreement with data presented in Figure 4D. In conclusion, by using the PRESTO-Tango and IP3 accumulation assays, we found that 3-hydroxydecanoate is predominantly an agonist for the MCFA receptor GPR84, and that the GPR84-induced signaling is mediated through Gα_i_.

### Other T2D-associated 3-hydroxy MCFAs are GPR84 agonists

As shown in Figure 5A and described previously, GPR84 can bind to MCFAs of several lengths, as well as to 3-hydroxy MCFAs.^23^^,^^29^ Interestingly, we found that postprandial levels of the 3-hydroxy MCFA 3-hydroxymyristate (3-OH-C14) were also associated with fasting glucose, HbA1c, and HOMA-IR, and that postprandial levels of 3-hydroxyoctanoate (3-OH-C8) were positively associated with fasting glucose and HbA1c.^53^ Using the IP3 accumulation assay, we observed that 3-hydroxyoctanoate was not a GPR84 agonist (Figure 5D, Table S1), but that 3-hydroxylaurate, with two additional carbons, was a GPR84 agonist and appeared more potent than laurate (Figure 5E). Similarly, 3-hydroxymyristate was also a more potent GPR84 agonist compared with myristate (Figure 5F). Overall, this shows how slight modifications of a metabolite can change the signaling capabilities through a GPCR, potentially leading to divergent functional responses of the agonists further downstream. Furthermore, it shows that a number of T2D-associated metabolites are GPR84 agonists.

### 3-Hydroxydecanoate mediates neutrophil migration through GPR84 and Gα_i_

**Figure 6:**
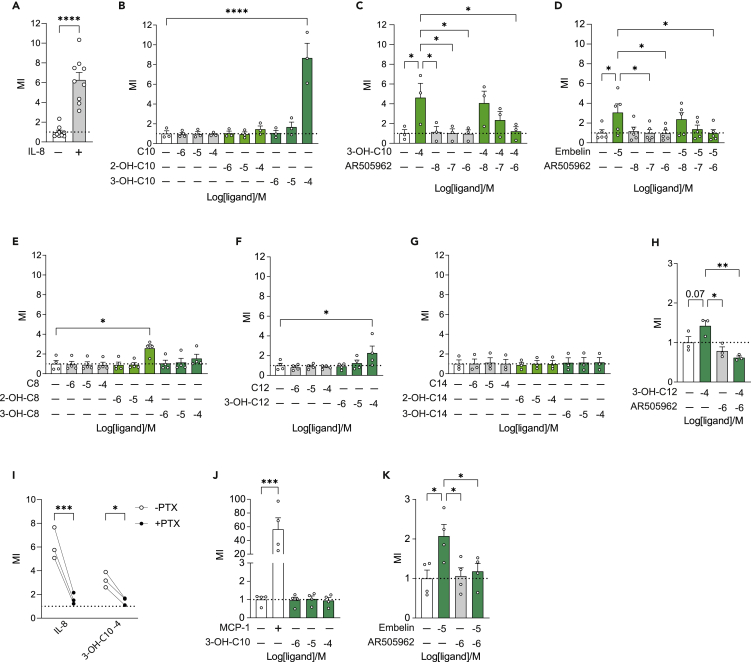
3-Hydroxydecanoate mediates neutrophil migration through GPR84 and Gα_i_ (A) Migration of human primary neutrophils toward 10 ng/mL IL-8 or DMSO vehicle (N = 9). (B) Migration of human primary neutrophils toward 3-hydroxydecanoate (3-OH-C10), 2-hydroxydecanoate (2-OH-C10), and decanoate (C10) (N = 3). (C and D) Migration of human primary neutrophils toward (C) 3-hydroxydecanoate or (D) embelin in the absence and presence of increasing concentrations of the GPR84 antagonist AR505962 (N = 3–5). (E–G) Migration of human primary neutrophils toward (E) octanoate (C8), (F) laurate (C12), or (G) myristate (C14) as well as their 2- and 3-hydroxy derivatives (N = 3–4). (H) Migration toward 3-hydroxylaurate (3-OH-C12) in the absence or presence of 1 μM (−6) AR505962 (N = 3). (I) Paired comparison of MI toward IL-8 or 3-hydroxydecanoate after pre-incubation of human primary neutrophils with or without 200 ng/mL PTX for 16 h (N = 3). (J) Migration of the human monocyte cell line THP-1 toward 20 ng/mL MCP-1 or 3-hydroxydecanoate (N = 4). (K) Migration of THP-1 cells toward embelin in the absence or presence of the GPR84 antagonist AR505962 (N = 4). Data are shown as mean migration indexes (MI) ± SEM MI was calculated as the relative migration of samples compared to the vehicle control. ∗p < 0.05, ∗∗p < 0.01, ∗∗∗p < 0.001, ∗∗∗∗p < 0.0001. p values were determined by two-tailed Mann-Whitney test (A), 1-way ANOVA followed by Dunnett’s multiple comparisons test (B–H and J–K), and two-way repeated measures ANOVA followed by Sidak’s multiple comparison test (I). See also Figure S6.

Since 3-hydroxydecanoate i.p. dosing gave rise to increased tissue inflammation and immune cell infiltration *in vivo*, and 3-hydroxydecanoate was able to mediate migration of murine neutrophils *in vitro*, we next investigated if 3-hydroxydecanoate could mediate migration of human peripheral neutrophils. Using a Transwell migration assay, we found that 3-hydroxydecanoate, as well as the positive control IL-8,^74^ induced potent migration of neutrophils (Figures 6A and 6B). In contrast, neither 2-hydroxydecanoate (2-OH-C10) nor decanoate induced migration (Figure 6B). Since 3-hydroxydecanoate is a GPR84 agonist and GPR84 is known to mediate migration of neutrophils and monocytes,^29^^,^^31^^,^^33^ we next investigated if the 3-hydroxydecanoate-mediated migration was GPR84 dependent. Importantly, GPR84 was expressed in both human and murine neutrophils, albeit at low levels (Figures S6A and S6B), suggesting that GPR84 could be involved in the migration. In agreement, we observed that the GPR84 antagonist AR505962 completely inhibited migration induced by 3-hydroxydecanoate (Figure 6C) as well as the GPR84 reference agonist embelin (Figure 6D). Since 2-hydroyxdecanoate and decanoate did not mediate migration of human neutrophils (Figure 6B), although they are GPR84 agonists (Figure 5B), further emphasize that the presence and location of the hydroxy group greatly affect the downstream effects of an agonist in a biological system.

Since other fatty acids with or without hydroxy groups also activated GPR84, we next investigated the migratory effect to these fatty acids. Interestingly, octanoate did not mediate migration (Figure 6E), although it activates GPR84 (Figure 5D); and neither did 3-hydroxyoctanoate (3-OH-C8). Conversely, 2-hydroxyoctanoate (2-OH-C8) which does not activate GPR84, did stimulate migration (Figure 6E), indicating that this migratory response is mediated though a different pathway. To our surprise, neither laurate (C12) nor myristate (C14), both GPR84 agonists (Figures 5A, 5E, and 5F), induced neutrophil migration (Figures 6F and 6G), supporting the notion that the presence of a hydroxy group is important for the migratory response. In agreement, the GPR84 agonist 3-hydroxylaurate (3-OH-C12) did stimulate migration (Figure 6F), while the GPR84 agonist 3-hydroxymyristate (3-OH-C14) did not (Figure 6G), indicating that also the chain length is important for the migratory response. Importantly, AR505962 also inhibited migration mediated by 3-hydroxylaurate (Figure 6H), suggesting that migration of neutrophils toward 3-hydroxydecanoate, embelin, and 3-hydroxylaurate are all mediated by GPR84. In summary, the presence and location of a hydroxy group as well as the acyl chain length were important for the migratory effect. Finally, our results indicate biased signaling of different GPR84 agonists, leading to divergent downstream cellular effects.

Cellular migration has been reported to be mediated through Gα_i_,^29^ and therefore we next investigated if 3-hydoxydecanoate-mediated migration was dependent on Gα_i_-signaling. To this end, we pre-incubated neutrophils with or without PTX and investigated, in a paired fashion, if the migratory capacity of each donor’s neutrophils changed in response to PTX. Pre-treatment with PTX resulted in significant inhibition of migration toward IL-8 and 3-hydroxydecanoate (Figure 6I), with the MI after PTX treatment almost reaching the baseline level of 1, which clearly show that the migration was indeed mediated through Gα_i_.

### 3-Hydroxydecanoate does not mediate monocyte migration

Finally, to investigate if the cellular bias in migratory response observed in murine neutrophils and monocytes (Figure 3) was replicated in human cells, we tested if the human monocyte cell line THP-1 could migrate toward 3-hydroxydecanoate in a Transwell migration assay. Importantly, the THP-1 cells did express *GPR84* (Figure S6C), making it possible that GPR84 could induce effects in this cell line. However, in agreement with the murine monocyte data (Figure 3L), 3-hydroxydecanoate did not stimulate migration of the THP-1 cells (Figure 6J). In contrast, the GPR84 reference agonist embelin stimulated THP-1 migration and was inhibited by AR505962 (Figure 6K), indicating that the receptor is present and able to induce migration, as also previously reported.^29^ Overall, this indicates a cellular bias in effects of 3-hydroxydecanoate, which is conserved between mice and humans.

## Discussion

Metabolomics has proven an important tool to identify potential disease-mediating metabolites. Here, we found that circulating levels of 3-hydroxydecanoate were increased in patients with T2D and that administration of 3-hydroxydecanoate to mice increased fasting serum insulin, induced tissue inflammation, and immune cell infiltration in iWAT and eWAT. *In vitro*, we confirmed that 3-hydroxydecanoate is a GPR84 agonist^29^^,^^54^ and demonstrated that 3-hydroxydecanoate mediates migration of murine and human neutrophils through GPR84-Gα_i_ signaling. We also demonstrate that chain length, hydroxylation, and position of the hydroxylation are essential for specificity of the signaling. Taken together, these findings suggest that 3-hydroxydecanoate may contribute to modulation of metabolic and inflammatory processes in T2D.

Here, we observed increased circulating levels of 3-hydroxydecanoate in individuals with T2D compared to obese controls without T2D. In agreement with our findings, a recent study also observed that 3-hydroxydecanoate levels were higher in obese individuals with T2D compared to healthy individuals^8^ and Al-Sulaiti et al. demonstrated that levels of 3-hydroxydecanoate, and also 3-hydroxyoctanoate, 3-hydroxylaurate, and 3-hydroxymyristate, increased with insulin resistance and T2D.^75^ We observed larger differences of 3-hydroxydecanoate between the groups following the MMT compared to fasting, suggesting that the differences in 3-hydroxydecanoate levels between T2D and controls could be due to modified absorption or metabolism of 3-hydroxydecanoate. Yet, the source of 3-hydroxydecanoate, as well as its regulatory mechanisms *in vivo*, is still unclear. However, 3-hydroxydecanoate has been found in milk and dairy products,^76^ implicating a potential dietary source in humans. We did not find 3-hydroxydecanoate in the different diets fed to mice, nor in the eWAT. However, it was present in plasma and the liver, and the plasma levels were higher after feeding HFD compared to chow, suggesting that 3-hydroxydecanoate is endogenously produced in mice and that the production is regulated by the type of diet, or that HFD is enriched in a precursor. The presence of 3-hydroxydecanoate in the liver could suggest production by the liver, which is supported by the finding that 3-hydroxydecanoate can be produced during mitochondrial β-oxidation.^55^ However, we cannot exclude that the metabolite is produced in adipose tissue and rapidly secreted, and was thus not be measured in the tissue. In addition, plasma 3-hydroxydecanoate decreased in response to refeeding after fasting, which could further support the β-oxidation origin. Finally, GF mice have increased fatty acid oxidation in peripheral tissues,^77^ and plasma 3-hydroxydecanoate levels were indeed higher in GF mice compared to CONV-R mice, further supporting β-oxidation as a source of 3-hydroxydecanote. Furthermore, Gram-negative bacteria in the gut utilize 3-hydroxydecanoate for LPS biosynthesis.^78^ Overall, our data support that 3-hydroxydecanoate may be produced as an intermediate molecule in mitochondrial β-oxidation in the liver and that its bioavailability may be influenced by the gut microbiota.

Administration of 3-hydroxydecanoate to mice resulted in increased fasting serum insulin levels, but this was not precipitated into impaired glucose tolerance. Importantly, 3-hydroxydecanoate promoted tissue inflammation and immune cell migration in both iWAT and eWAT, which was associated with increased numbers of neutrophils in iWAT and neutrophils as well as macrophages in eWAT. Surprisingly, we observed fewer immune cells in the liver after 3-hydroxydecanoate administration, which require further examination in the future. A potential explanation for the lack of effect on macrophage recruitment to iWAT could be that neutrophil recruitment precedes that of macrophages, as previously reported.^79^^,^^80^ These data suggest that the inflammation in eWAT and iWAT was not sufficient to induce impaired glucose tolerance, or that more chronic treatments would have been required to observe this.

*In vitro*, we observed that 3-hydroxydecanoate specifically promoted neutrophil, but not monocyte migration. We further identified that human neutrophil migration toward 3-hydroxydecanoate was mediated through GPR84-Gα_i_, in line with previous reports that GPR84 agonists, e.g. embelin, 6-OAU, ZQ-16, and 3-hydroxylaurate, mediate immune cell migration,^29^^,^^31^^,^^33^^,^^38^^,^^81^ and that it can be mediated through Gα_i_.^29^ PTX treatment reduced the 3-hydroxydecanoate-induced MI, confirming that this migration likewise was mediated through Gα_i_. The finding that the GPR84 antagonist AR505962 completely blocked the migration toward 3-hydroxydecanoate and 3-hydroxylaurate indicates that GPR84 indeed is required for migration toward these fatty acids. This further suggests that the lack of migration toward other GPR84 agonists, e.g. decanoate and laurate, is due to differences in intracellular signaling, i.e. biased signaling. In support of this, differential cellular effects have previously been described for two synthetic GPR84 agonists: DL-175 is biased toward G protein signaling, whereas 6-OAU elicits both G protein and β-arrestin signaling. However, only 6-OAU stimulates migration,^82^ suggesting that specific intracellular signaling pathway(s) are important for the downstream cellular events. Molecular modeling and single amino acid substitutions have identified specific amino acids that are involved in the binding of different GPR84 ligands.^83^^,^^84^^,^^85^ Interestingly, 6-OAU, DL-175, and also the GPR84 agonist 2-HTP interact with different amino acid residues in the GPR84 binding sites.^86^ It is thus possible that the differences in migratory effect of the GPR84 agonists observed here could be due to different binding properties of the agonists, leading to biased signaling where 3-hydroxydecanoate induces cell migration likely independent of β-arrestin signaling. However, we cannot exclude that some other unknown mechanism may be involved, making GPR84 a required factor, but not sufficient for migration, including that 3-hydroxydecanoate can be taken up by cells and elicit metabolic functions.

We also confirmed that fatty acid-mediated signaling through GPR84 depends on the length of the acyl chain, and that a single hydroxy group on a fatty acid can greatly affect signaling potency as well as downstream cellular effects.^29^ This was reflected in the observation that 3-hydroxydecanoate, but not the reported endogenous agonist for GPR84, decanoate,^23^ induced neutrophil migration in our study. Similarly, 3-hydroxydecanoate and other 3-hydroxy MCFAs elicited immune responses, e.g. ROS production and expression of plant defense genes, in *Arabidopsis* plants, and these responses depended on the length of the acyl chain as well as the presence of a hydroxy group.^56^ Although decanoate and 3-hydroxydecanoate both signal through GPR84 in the *in vitro* GPCR signaling assays tested, they differ *in vivo*: Decanoate improves glucose sensitivity in mice,^87^ is decreased in T2D,^7^ and increased after Roux-en-Y gastric bypass.^88^ In contrast, we found that 3-hydroxydecanoate increases fasting insulin in mice and is increased in T2D. Apart from GPR84, decanoate also signals through GPR40^73^ and can directly activate PPARγ^89^; while 3-hydroxydecanoate, in our study, appears to be a more selective agonist for GPR84, yet with a potential minor signaling capacity through GPR40. This suggests that GPR84 may be tightly linked to regulation of metabolism and that 3-hydroxydecanoate may be a more potent endogenous agonist for GPR84 than decanoate. Taken together, knowledge of differential *in vitro* and *in vivo* effects of decanoate and 3-hydroxydecanate, including the suggested biased signaling of the agonists, is of great relevance for potential drug design, as targeting of specific signaling pathway(s) can allow for more precise cellular responses as well as reduce the risk of unwanted side effects. Yet, the complete delineation of the signaling events elicited by decanoate, 3-hydroxydecanoate, and other GPR84 agonists is yet to be fully described.

In agreement with a potential pro-inflammatory role of 3-hydroxydecanoate signaling through GPR84, antagonists to this receptor were previously demonstrated to inhibit neutrophil migration and inflammation in acute liver injury^38^ as well as in an inflammatory bowel disease model in mice.^37^ Furthermore, GPR84 is predominantly considered a pro-inflammatory receptor capable of inducing expression of pro-inflammatory genes and release of pro-inflammatory cytokines in immune cells.^23^^,^^25^^,^^29^^,^^30^^,^^31^ Thus, it is possible that the 3-hydroxydecanoate-mediated neutrophil migration and tissue inflammation we observed could be mediated by GPR84 signaling. In agreement, GPR84 KO mice display decreased *Ccl2* expression in adipose tissues^35^, and exhibit improved HOMA-IR on an HFD, suggesting a role for GPR84 not only in inflammation but also in insulin resistance and glycemic control.^35^ In addition to being highly expressed in immune-related tissues and cells,^23^^,^^24^^,^^25^ *Gpr84* is also expressed in adipose tissues, skeletal muscle, pancreas, and the liver.^34^ Skeletal muscle from *Gpr84* KO mice had increased triglyceride content,^34^ and livers from *Gpr84* KO mice had increased triglyceride content after feeding an MCFA-enriched diet,^36^ suggesting a role of GPR84 also in lipid metabolism. Furthermore, in murine pancreatic islets, the GPR84 agonist laurate stimulated insulin secretion.^34^ Additional studies using *Gpr84* KO mice or GPR84 antagonists are, therefore, needed in order to fully address if the *in vivo* effects observed upon 3-hydroxydecanoate dosing here are indeed mediated through GPR84.

One limitation is that we only included obese individuals with a BMI above 35 kg/m^2^ in our study and have no information if 3-hydroxydecanoate is similarly higher in lean individuals with T2D.^52^ Second, we were unable to breed and obtain viable *Gpr84* KO mice, thus preventing us from confirming if the effects of 3-hydroxydecanoate were indeed mediated through GPR84 *in vivo*. Finally, while our *in vitro* data show GPR84-dependent neutrophil migration, we cannot exclude the possibility that 3-hydroxydecanoate may also elicit GPR84-independent effects, e.g. by being taken up by cells and stimulate cellular events by virtue of its metabolite nature.

In summary, we characterized the *in vitro* and *in vivo* effects of 3-hydroxydecanoate, a metabolite enriched in T2D. We found that 3-hydroxydecanoate mediates neutrophil migration through GPR84-Gα_i_ and increases WAT inflammation as evident by increased neutrophil and macrophage recruitment as well as increased inflammatory gene expression, which may contribute to the chronic inflammation and high insulin levels observed in T2D. Accordingly, better understanding of GPR84-mediated roles of 3-hydroxydecanoate in T2D may result in new approaches to block its signaling at tissue level to improve T2D.

### Limitations of the study

There are some limitations in the study. First, we only included obese individuals with a BMI above 35 kg/m^2^ in our study and have no information if 3-hydroxydecanoate is similarly higher in lean individuals with T2D.^52^ Second, we were unable to breed and obtain viable *Gpr84* KO mice, thus preventing us from confirming if the effects of 3-hydroxydecanoate were indeed mediated through GPR84 *in vivo*. Finally, while our *in vitro* data show GPR84-dependent neutrophil migration, we cannot exclude the possibility that 3-hydroxydecanoate may also elicit GPR84-independent effects, e.g. by being taken up by cells and stimulate cellular events by virtue of its metabolite nature.

## STAR★Methods

### Key resources table

**Table:** 

REAGENT or RESOURCE	SOURCE	IDENTIFIER
**Antibodies**		

Rat anti-mouse Ly-6G	Biolegend	Cat#127601; RRID: AB_1089179
Rabbit monoclonal anti-mouse CD68/SR-D1 clone 2449D	R&D Systems	Cat#MAB101141
Goat anti-human polyclonal anti-perilipin	Abcam	Cat#ab61682; RRID: AB_944751
Alexa Fluor 555 donkey anti-rat	Invitrogen	Cat#A48270; RRID: AB_2896336
Alexa Fluor 647 donkey anti-rabbit	Invitrogen	Cat#A31573; RRID: AB_2536183
Alexa Fluor 488 donkey anti-goat	Invitrogen	Cat#A11055; RRID: AB_2534102
RNAscope® Protease Plus, Protease IV	ACD	Cat#322331

**Chemicals, peptides, and recombinant proteins**		

Zaprinast	Sigma	Cat#Z0878
(±)-3-Hydroxydecanoic acid	Sigma	Cat#H3648
3-Hydroxydecanoic acid	Larodan	Cat#14–1003
Capric acid	Sigma	Cat#21409
Embelin	Sigma	Cat#E1406
(±)-2-Hydroxydecanoic Acid	Toronto Research Chemicals	Cat#H235135
Dodecanoic acid	Sigma	Cat#L4250
(±)-3-Hydroxydodecanoic Acid	Toronto Research Chemicals	Cat#H943755
Myristic acid	Sigma	Cat#M3128
D,L-α-Hydroxy Myristic Acid	Toronto Research Chemicals	Cat#H948500
(±)-3-Hydroxytetradecanoic Acid	Toronto Research Chemicals	Cat#H956780
Sodium butyrate	Sigma	Cat#B5887
Sodium hexanoate	Sigma	Cat#C4026
Octanoic acid	Sigma	Cat#C5038
2-hydroxyoctanoic acid	Sigma	Cat#H7396
3-hydroxyoctanoic acid	Sigma	Cat#H3898
Disodium sebacate	Sigma	Cat#CDS000582
3-Hydroxy Sebacic Acid	Toronto Research Chemicals	Cat#H953770
AR505962	Arena Pharmaceuticals	N/A
TAK-875	Merck	Compound 9a in Negoro et al., 2010^70^
Barbadin	Toronto Research Chemicals	Cat#B118250
PTX	Sigma	Cat#P2980
YM-254890	FUJIFILM Wako Chemicals U.S.A. Corporation	Cat#257–00631
Linoleic acid	Sigma	Cat#L2376
Merck B	Merck	Example 209 in Shi et al., 2010^104^
AR231783	Arena Pharmaceuticals	N/A

**Critical commercial assays**		

Ultra Sensitive Mouse Insulin ELISA Kit	Crystal Chem	Cat#90080
Monocyte Isolation Kit (BM)	Miltenyi Biotec	Cat#130-100-629
CellTiter-Glo®	Promega	Cat#G7570
Boyden style Corning® HTS Transwell® 96 well system, 3 μm	Sigma	Cat#CLS3385
Boyden style Corning® HTS Transwell® 96 well system, 5 μm	Sigma	Cat#CLS3388
Steadylite plus Reporter Gene Assay System	PerkinElmer	Cat#6066759
LS Columns	Miltenyi Biotec	Cat#130-042-401
YSi Poly-L-Lysine coated beads	PerkinElmer	Cat#RPNQ0010
^3^H-myo-inositol	PerkinElmer	Cat#NET114A005MC
V-PLEX Pro-inflammatory Panel 1 Mouse Kit	MSD	Cat#K15048D

**Experimental models: Cell lines**		

Human: HT29 cells	ATCC	Cat#HTB-38; RRID: CVCL_0320
Monkey: COS cells	ATCC	Cat#CRL-1651; RRID: CVCL_0224
Human: THP-1 cells	Dr. Søren Skov	N/A
Human: HTLA cells	Dr. Wesley Kroeze and Dr. Bryan Roth	Barnea et al., 2008^68^; Kroeze et al., 2015^69^

**Experimental models: Organisms/strains**		

Mouse: Swiss Webster	Taconic	Cat#SW

**Oligonucleotides**		

Primers for qRT-PCR, see Table S2	This paper	N/A

**Recombinant DNA**		

PRESTO-Tango GPCR kit	AddGene	Cat#1000000068
Gα_Δ6qi4myr_	Kostenis, 2001^72^	N/A
Receptor plasmid, human GPR84	Arena Pharmaceuticals	N/A
Receptor plasmid, human GPR40 (FFA1)	Ekberg et al., 2016^90^	N/A
Receptor plasmid, human GPR120 (FFA4)	Ekberg et al., 2016^90^	N/A
Receptor plasmid, human GPR109B (HCA3)	Origene	N/A
Receptor plasmid, human FFA2 (GPR43)	Nohr et al., 2013^91^	N/A
Receptor plasmid, human FFA3	Nohr et al., 2013^91^	N/A
Receptor plasmid, human HCA1 (GPR81)	Origene	N/A
Receptor plasmid, human HCA2 (GPR109A)	Origene	N/A
Receptor plasmid, human GPR91	Trauelsen et al., 2017^92^	N/A
Receptor plasmid, human GPR142	Rudenko et al., 2019^93^	N/A
Receptor plasmid, human GPR35	Arena Pharmaceuticals	N/A
Receptor plasmid, human FPR1	Origene	N/A
Receptor plasmid, murine GPR84	Origene	Cat#MC#203180

**Software and algorithms**		

GraphPad Prism 9.3.0	GraphPad	https://www.graphpad.com/
MzMine 2.53	Pluskal et al., 2010^94^	http://mzmine.github.io/
RTCA Software Pro 2.3.2	ACEA Biosciences	https://www.agilent.com/en/product/cell-analysis/real-time-cell-analysis/rtca-software/rtca-software-pro-741236
ImageJ 1.53o	ImageJ	https://imagej.nih.gov/ij/
ZEN blue software	Zeiss	https://www.zeiss.com/microscopy/int/products/microscope-software.html

### Resource availability

#### Lead contact

Data reported in this paper will be shared by the lead contact upon reasonable request to the lead contact, Fredrik Bäckhed (Fredrik.Backhed@wlab.gu.se). Clinical data will be shared by the clinical PI of the BARIA study (m.nieuwdorp@amsterdamumc.nl).

#### Materials availability

This study did not generate new unique reagents.

### Experimental models and subject details

#### Patients and metabolomics

Participants in the BARIA study were recruited as described,^52^ with a total of 106 (84 women and 22 men) individuals included. Those with T2D tended to be slightly older (47.1 ± 10.1 vs 45.4 ± 10.0 years) and enriched in men (27.3 vs 19%) while the differences did not reach statistical differences (Table 1). The study was performed in accordance with the Declaration of Helsinki and was approved by the Ethical Review Board of the Academic Medical Center, Amsterdam (approval code: NL55755.018.15). All participants provided written informed consent. Anthropometric and metabolic characteristics, including a 2 h MMT, were assessed as previously described.^52^^,^^53^ Fasting and MMT plasma samples taken prior to bariatric surgery were analyzed by METABOLON (Morisville, NC, USA) using ultra high-performance liquid chromatography coupled to tandem mass spectrometry (LC-MS/MS) untargeted metabolomics.^5^ The full dataset is presented in Li et al.^53^ The log transformed values of 3-hydroxydecanoate in plasma at the fasting and postprandial states were plotted as boxplots using GraphPad Prism.

For isolation of human peripheral neutrophils, the donors were healthy males between 20 and 40 years of age.

#### Animal housing and husbandry

Germ-free (GF) and conventionally raised (CONV-R) Swiss Webster mice were obtained from Taconic (Taconic #SW) and thereafter bred in-house. GF mice were maintained and bred in plastic film isolators and sterility was assessed by PCR. CONV-R mice were housed in a specific pathogen free environment. All mice had free access to autoclaved rodent chow diet (Lab diet #5010) and water *ad libitum*. All animal experiments were approved by the Animal Experiments Inspectorate under the Danish Ministry of Food, Agriculture and Fisheries and were performed according to the institutional guidelines at University of Copenhagen, Denmark in a fully AAALAC accredited facility. The mice used in experiments were male and between 7 and 14 weeks when used. The specific ages in the individual experiments are indicated in the description of each experiment below.

#### Cell lines

The human, female colon cancer cell line HT29 (ATCC #HTB-38) was maintained in McCoy’s 5A medium (Sigma #M8403) supplemented with 10% v/v FBS (Sigma #F7524), 200 IE/mL penicillin (SSC, UCPH), 50 μg/mL streptomycin (SSC, UCPH) and 1.5 mM L-glutamine (SSC, UCPH) in a humidified atmosphere at 37°C and 5% CO_2_.

The monkey COS-7 cells (ATCC Cat#CRL-1651) were maintained in DMEM GlutaMAX (Gibco #21885) supplemented with 10% v/v FBS (Sigma #F7524), 200 IE/mL penicillin (SSC, UCPH) and 50 μg/mL streptomycin (SSC, UCPH) in a humidified atmosphere at 37°C and 10% CO_2_.

The engineered human, female HEK293 cell line HTLA was kindly provided by Dr. Wesley Kroeze and Dr. Bryan Roth, UNC School of Medicine, North Carolina, USA. The HTLA cells were maintained in DMEM (Gibco #21885–025) supplemented with 10% v/v FBS (Sigma #F7524), 200 IE/mL penicillin (SSC, UCPH), 50 μg/mL streptomycin (SSC, UCPH), 2 mM L-glutamine (SSC, UCPH), 2 μg/mL puromycin (Sigma, #P9620) and 100 μg/mL hygromycin B (Thermo Fisher #10687010) and cultured in a humidified atmosphere at 37^o^C and 5% CO_2_.

The human, male monocyte cell line THP-1 was kindly provided by Dr. Søren Skov, University of Copenhagen, and maintained in RPMI-1640 medium with GlutaMAX (Gibco #61807) supplemented with 10% v/v FBS (Sigma, #F7524), 200 IE/mL penicillin (SSC, UCPH) and 50 μg/mL streptomycin (SSC, UCPH), and cultured in a humidified atmosphere at 37°C and 5% CO_2_.

### Method details

#### Fasting-refeeding

7-12-week-old male CONV-R Swiss Webster mice (N = 14) were fasted overnight for 16 h, but still had access to drinking water *ad libitum*. A baseline blood sample was taken by retro-orbital sinus bleeding into VITREX® EDTA-coated capillary tubes (VWR #MODU164213). Thereafter, mice were allowed *ad libitum* access to autoclavable rodent chow diet (Lab diet #5010), and subsequent blood samples were taken one, two and 4 h after refeeding. All blood samples were centrifuged at 5,000x*g* for 5 min, plasma was separated, snap-frozen and stored at −80°C until metabolomics analysis.

#### Plasma collection in mice

To compare levels of 3-hydroxydecanoate in GF and CONV-R Swiss Webster mice, male mice of 13–14 weeks of age (N = 5–6 mice/group) were fasted for 4 h. Blood was collected by retro-orbital sinus bleeding into VITREX® EDTA-coated capillary tubes (VWR #MODU164213). Plasma was separated after centrifugation at 5,000x*g*, snap-frozen and stored at −80°C until metabolomics analysis.

#### HFD feeding

To compare levels of 3-hydroxydecanoate in CONV-R Swiss Webster mice fed a chow or HFD, male mice of 10–11 weeks of age (N = 6 mice/group) were fed a standard chow diet or HFD (research Diets #D12492i) with 60% energy from fat for two weeks. The mice were then fasted for 4 h, anesthetized using isoflurane (Baxter #EAAG9623C) and blood was collected from vena cava into Microvette® 500 K3 EDTA-coated capillary tubes (Sarstedt #20.1341). Plasma was separated after centrifugation (10,000xg for 5 min), snap-frozen and stored at −80°C until metabolomics analysis. Epididymal fat pads and the liver were harvested and snap-frozen in liquid nitrogen for metabolomics analysis.

#### Targeted analysis of 3-hydroxydecanoate

For plasma metabolomics analysis, 20 μL plasma was taken for extraction, and 10 μL of each sample was taken for the pooled sample, which was subsequently aliquoted in 20 μL aliquots10 μL 3-hydroxytridecanoic acid (2 mg/L) (Cayman Chemicals #22689), which served as internal standard, and 100 μL methanol (Honeywell #1426) were added to all samples. After vortexing, suspension was left to precipitate for 30 min on ice. Next, extracts were centrifuged at 10,000 rpm for 3 min at 4°C, and 100 μL supernatant was collected in LC vials (Thermo Scientific #03-FISV) and left to evaporate to dryness under a stream of nitrogen. Dried extracts were reconstituted in 40 μL methanol. In order to identify and quantify target analytes, a dilution series of authentic standards was prepared in methanol and extracted the same way as samples.

Tissue and food samples were cryo-pulverized using CP02 cryoPREP Automated Dry Pulverizer (Covaris #500000) and TT05 tissue bags (Covaris #520072). For each 10 mg cryogenic tissue or food powder, 190 μL methanol was added along with 10 μL 3-hydroxytridecanoic acid internal standard (2 mg/L). Suspensions were vortexed and sonicated for 15 min in ice-cold water, and next left to precipitate on ice for 15 min. Extracts were centrifuged for 3 min at 10,000 rpm at 4°C, and 200 μL supernatant was used for evaporation as described above; and 100 μL was taken to create pooled samples which were prepared in the same way as the other samples. After evaporation, samples were reconstituted with 50 μL of methanol, vortexed and kept at −20°C until injection. 50 μL of each native standards calibration point was added to 10 μL internal standard and 140 μL methanol. After that, it was evaporated and reconstituted in the same way as samples.

All samples were analyzed by LC/MS. Agilent 1290 Infinity II Liquid chromatograph (Agilent) was used for the chromatographic separation, and Tims TOF Pro mass spectrometer (Bruker) was used for detection. Separation was made with Waters HSS T3 10 cm × 2.1 × 1.8 μm column (Waters #186003539). Mobile phase A was water with added 0.1% formic acid (Thermo Scientific #A117-50) while mobile phase B was composed of acetonitrile (Honeywell #14261) and isopropanol (Honeywell #34965) (V:V = 3:1) with 0.1% formic acid. Mobile phase gradient started with 3% of mobile phase B, and thereafter increased to 100% mobile phase B in 9 min. Next, it was left stable for 5 min and subsequently re-equilibration to initial conditions. Column temperature was kept at 40°C and injection volume was 10 μL. Acquisition was performed in full scan negative mode in the mass range from 50 to 500 M/z.

Data extraction was made with MzMine 2.53^94^ in a targeted way. Extracted areas under the peak were normalized with area of internal standard and compared to calibration curves for quantification. Concentration calculations were done in Excel, and figures were prepared in GraphPad Prism. Concentrations in plasma are expressed as nM and in tissue and food as nmol/kg of tissue.

#### Administration of 3-hydroxydecanoate *in vivo*

Male CONV-R Swiss Webster mice of 12 weeks of age were fasted for 4 h, weighed and randomized in a weight-matched manner into two groups (N = 6 mice/group). In the morning, mice were injected i.p. with 25 mg/kg 3-hydroxydecanoate (Larodan #14–1003) dissolved in 5% v/v DMSO (Sigma #D5879) in 0.9% saline (Mediq Danmark #B230553) for seven days. The vehicle group received 5% v/v DMSO in saline. Based on the 3-hydroxydecanoate (or vehicle) stock solution, the dosing volume was 5.32 μL/g body weight, resulting in a dose of 0.29 mg/g body weight DMSO. On day eight, the mice were fasted for 4 h, anesthetized with isoflurane (Baxter #EAAG9623C), and the vena cava blood was collected into Microvette® 500 K3 EDTA-coated capillary tubes (Sarstedt #20.1341). Blood samples were centrifuged (10,000xg for 5 min) and separated plasma was snap-frozen and stored at −80°C until cytokine measurements. Epididymal and inguinal fat pads and the liver were harvested and snap-frozen in liquid nitrogen for RNA isolation. In addition, the epididymal and inguinal fat pads and liver were harvested, placed in the cassettes and stored in 4% formaldehyde (VWR #9713.5000) for two days before embedding in paraffin.

#### Histology

After harvest and storage of tissues in 4% formaldehyde (VWR #9713.5000) for two days, the tissues were next processed using the Excelsior AS (Thermo Scientific) and embedded in paraffin blocks (Hounisen #2280.6060). Sections of 4 μm thickness were next cut from three different block depths (with 100 μm distance between them) using the Microm Ergostar HM200 microtome (ZEISS) and placed on Superfrost^TM^ microscope slides (Thermo Scientific #J1800AMNZ). Each section contained eWAT, iWAT, liver and spleen. From each mouse, one section from each depth was stained. Briefly, slides were deparaffinised in xylene (VWR #28973.363), followed by decreasing concentrations of ethanol from 99% (VWR #28973.375), 96% (VWR #20824.365) to 70% (VWR #83801–360). After washing in PBS (SSC, UCPH) supplemented with 0.1% Triton X-, antigen was retrieved by boiling for 10 min in sodium citrate buffer (10 mM sodium citrate (Merck #1.06432.1000) in water, 0.05% v/v TWEEN®20 (Sigma #P9416), pH 6.0), followed by cooling and treatment with protease IV (ACD #322331) for 10 min at 40°C. The slides were blocked in 2% BSA (Sigma #A7030) in PBS with 0.1% Triton for 1 h. Next, the slides were incubated over night with primary antibodies diluted in the blocking solution. The rat anti-mouse Ly-6G (Biolegend #127601) and rabbit monoclonal anti-mouse CD68/SR-D1 clone 2449D (R&D Systems #MAB101141) were diluted 1:1000, and the goat anti-human polyclonal anti-perilipin (Abcam #ab61682) was diluted 1:200. Next day, the slides were washed in PBS with 0.1% Triton and incubated with secondary antibodies for 1 h. The secondary antibodies Alexa Fluor 555 donkey anti-rat (Invitrogen #A48270), Alexa Fluor 647 donkey anti-rabbit (Invitrogen #A31573) and Alexa Fluor 488 donkey anti-goat (Invitrogen #A11055) were all diluted 1:800 in the blocking solution. Finally, the slides were washed, and mounted using Pro-Long Gold Antifade with DAPI (Thermo Fisher #P36941).

For imaging and quantification, five pictures from tissue in each section were taken on a Zeiss Axioobserver microscope (Zeiss) using the Axiocam 702 camera and the ZEN blue software (Zeiss), at 20× magnification. Tissue area in each image was calculated using ImageJ, the images were randomized, and the number of Ly6G^+^ neutrophils and CD68^+^ macrophages were counted in a blinded fashion without the counting person knowing whether the image was from a vehicle- or 3-hydroxydecanoate-treated mouse. The data is presented as density of Ly6G^+^ or CD68^+^ cells.

#### Cytokine analysis

Vena cava plasma levels of IL-1β, IL-6, KC/GRO and TNF-α were measured using the V-PLEX® Pro-inflammatory Panel 1 Mouse kit (Mesoscale Diagnostics #K15048D) according to the manufacturer’s instructions. All samples were measured in duplicates. The plates were analyzed on a MESO QuickPlex SQ 120 (Mesoscale Diagnostics) using the plate barcode, and data was analyzed using the MSD Discovery Workbench software version 4.0.12 (Mesoscale Diagnostics).

#### 3-Hydroxydecanoate dosing for ipGTT

Male CONV-R Swiss Webster mice of 10–13 weeks of age were weighed, MRI scanned and randomized in a weight-matched manner into two groups (N = 12–13 mice/group). Mice were injected i.p. with 25 mg/kg 3-hydroxydecanoate (Larodan #14–1003) dissolved in 5% v/v DMSO (Sigma #D5879) in saline (Mediq Danmark #B230553) for seven days. The vehicle group received 5% v/v DMSO in saline. Based on the 3-hydroxydecanoate (or vehicle) stock solution, the dosing volume was 5.32 μL/g body weight, resulting in a dose of 0.29 mg/g body weight DMSO. On day seven, after the last 3-hydroxydecanote or vehicle dosing, the mice were fasted for 4 h, followed by an ipGTT. The mice were dosed i.p. with 2 mg/kg glucose (Sigma #G7021) and blood glucose was measured using Bayer Contour XT glucometers (Bayer #84030970) and strips (Bayer #84030881). Tail bleedings for glucose and insulin measurements were taken 30 min before glucose dosing, and thereafter blood glucose was measured right before dosing (time point 0) and 15, 30, 60, 90 and 120 min after glucose dosing. Blood samples were centrifuged (5,000xg for 5 min) and separated serum was snap-frozen and stored at −80°C until insulin measurements were performed. Finally, the mice were scanned using the Bruker minispec MRI (Bruker) and euthanized.

#### Insulin measurement

Insulin concentration in mouse serum was measured using the Ultra Sensitive Mouse Insulin ELISA Kit (wide range assay, 0.1–12.8 ng/mL) from Crystal Chem (Crystal Chem #90080) according to the manufacturer’s instructions.

#### Plasmid isolation

Plasmids encoding GPCRs or Gα_Δ6qi4myr_^71^ were isolated from bacterial cultures grown in LB medium (SSC, UCPH) containing 100 mg/mL ampicillin (Sigma #A5354) or 50 mg/mL kanamycin (Sigma #K0254) depending on the antibiotic resistance gene present in each plasmid. Plasmids were purified using the NucleoBond Xtra Midi Plasmid DNA purification kit (Macherey Nagel #740410) according to the manufacturer’s instructions. DNA was dissolved in sterile water (Amgros #744128) and stored at −20°C. The DNA concentration and quality was measured on a NanoDrop ONE Spectrophotometer (Thermo Scientific).

#### Eurofins DiscoverX

The PathHunter β-arrestin assay relies on endogenous β-arrestin recruitment. β-arrestin is fused to an inactive deletion mutant of β-galactosidase, and the GPCR is fused to the deleted fragment of β-galactosidase. When the β-arrestin/β-galactosidase-complex binds to a GPCR, β-galactosidase becomes active and enzyme activity can be measured using chemiluminescence.

3-hydroxydecanoate at 100 μM was screened in the Eurofins DiscoverX orphanMAX and gpcrMAX panels using the PathHunter® β-Arrestin enzyme fragment complementation (EFC) technology. In the gpcrMAX panel, 3-hydroxydecanoate was tested in agonist and antagonist mode on 168 GPCRs. In the orphanMAX panel, 73 GPCRs were tested in agonist mode. According to the provided guidelines, the compound was considered an agonist, if it increased activity by >50% in the orphanMAX or >30% in the gpcrMAX. In the antagonist mode, an inhibition of >35% was indicative of the compound potentially acting as an antagonist.

#### xCELLigence

The xCELLigence assay was run in E-plate 96 PET (ACEA Biosciences #300600900) using the xCELLigence RTCA SP station (ACEA Biosciences). As a cell model we used the HT29 cell line, since this has previously been successfully used in label-free assay systems such as the xCELLigence or similar assays, including for the study of GPCRs.^95^^,^^96^^,^^97^^,^^98^

Initially, culture medium was added to all wells and spaces between wells in the E-plate to measure background in the SP station. The medium in each well was next replaced by 25,000 HT29 cells in 100 μL culture medium, and the cells were left to settle for 30–40 min. The plate was next mounted in the xCELLigence SP station for monitoring overnight with readings performed every hour. Next day, medium was replaced with pre-heated assay medium (McCoy’s 5A medium (Sigma #M8403) with 1% v/v FBS (Sigma #F7524), 200 IE/mL penicillin (SSC, UCPH), 50 μg/mL streptomycin (SSC, UCPH) and 1.5 mM L-glutamine (SSC, UCPH), and the E-plate was left in the SP station to equilibrate and read for another 2 h. Before compound addition, a normalization measurement was performed. For agonist treatment, compounds were added and readings were performed every 15 s for 21/2 hours. For antagonist mode, the cells were pre-incubated with 2 μM YM-254890 (FUJIFILM Wako Chemicals #257–00631) or 10 μM barbadin (Toronto Research Chemicals #B118250) for 15 min with readings performed every 15 s; followed by agonist stimulation as described. For PTX (Sigma #P2980) (200 ng/mL) treatment, the cells were pre-incubated for 16 h, followed by agonist stimulation as described. All compounds, except PTX which was supplied in a buffered aqueous glycerol solution, were prepared in 20% DMSO (Sigma #D5879). The final DMSO concentration of the wells was maintained at 0.2% in agonist mode and 0.4% in antagonist mode. The GPR35 agonist zaprinast^99^ (Sigma #Z0878) was used as a positive control, since previous and in-house studies had found it to function well in the xCELLigence assay with HT29 cells.^95^^,^^96^ All measurements were performed in triplicate.

Data was analyzed using the RTCA Software Pro (ACEA Biosciences) and visualized in GraphPad Prism. All cell indexes (CI) were normalized to the normalization reference (the last measurement before agonist addition), followed by normalization to the baseline CI (vehicle control). The peak response within the first 60 min after agonist addition was used for calculating dose-response curves and to assess the effect of inhibitors. EC50 values for the dose-response curves were calculated using nonlinear regression in GraphPad Prism.

#### IP3 accumulation assay

COS-7 cells were maintained in DMEM GlutaMAX (Gibco #21885) supplemented with 10% v/v FBS (Sigma #F7524), 200 IE/mL penicillin (SSC, UCPH) and 50 μg/mL streptomycin (SSC, UCPH) in a humidified atmosphere at 37°C and 10% CO_2_. For assays, 20,000 cells/well were seeded in poly-D-lysine (Sigma #P9620) coated clear 96 Well Cell Culture Plates (Costar #3599). After overnight incubation, the cells were transfected with plasmid DNA. For human GPCRs signaling through Gα_q_ (FFA1/GPR40,^90^ FFA2/GPR43^91^ and FFA4/GPR120^90^), the cells were transfected with GPCR construct only (40 μg/plate). For remaining human GPCRs not signaling through Gα_q_ (GPR109B/HCA3 (Origene), FFA3/GPR41,^91^ HCA1 (Origene), HCA2 (Origene), GPR91,^92^ GPR142,^93^ FPR1 (Origene), GPR35 (Arena Pharmaceuticals), and GPR84 (Arena Pharmaceuticals), as well as the murine GPR84 (Origene #MC#203180), the cells were transfected with GPCR construct (25 μg/plate) plus the chimeric G protein Gα_Δ6qi4myr_ (25 μg/plate plate).^72^ Gα_Δ6qi4myr_ consists of the Gα_q_ backbone, but the four C-terminal amino acid residues have been exchanged with those of Gα_i_. This allows the chimeric G protein to bind GPCRs that normally bind Gα_i_, but the intracellular signaling will be diverted toward IP3 accumulation.^72^

Transfection was performed using a calcium transfection protocol. Briefly, DNA was mixed with CaCl_2_ (SSC, UCPH) and TE buffer (SSC, UCPH). The mix was carefully dripped into an equal volume of 2x HBS buffer (SSC, UCPH) and incubated at room temperature (RT) for 45 min. Next, 10 mL/plate COS-7 culture medium and 300 μL chloroquine at 2 mg/mL (Sigma #C6628) in PBS was added, and 100 μL DNA-medium mix was added to each well with cells. After incubating for 5 h, the medium was replaced with standard COS-7 culture medium supplemented with 5 μL/mL ^3^H-myo-inositol (PerkinElmer #NET114A005MC) and left overnight at 37°C and 10% CO_2_. After incubation, the cells were washed in HBSS (Gibco #14025–050) followed by HBSS with 10 mM LiCl (SSC, UCPH). Next, the cells were stimulated with compounds (see key resources table) and incubated for 2.5 h at 37°C. The cells were lysed on ice with 50 μL/well 10 mM formic acid (SSC, UCPH) for 30 min; and 20 μL lysates were added to white 96 well plates (Sigma #CLS3917) containing 80 μL/well YSi Poly-L-Lysine coated beads (PerkinElmer #RPNQ0010). Finally, the plates were shaken, centrifuged and counted on a Micro-Beta^2®^ (PerkinElmer) with 4 h’ delay. All measurements were performed in duplicate. The data was normalized to the baseline and maximum response of the reference agonist for each receptor, and EC50 values were calculated using nonlinear regression in GraphPad Prism.

#### PRESTO-Tango

The PRESTO-Tango is independent of endogenous signaling, because it relies solely on an engineered signaling pathway. Each GPCR is coupled to the C terminus of the V_2_ vasopressin receptor, which binds β-arrestin strongly and thus allows binding of β-arrestin to GPCRs that may not bind it in their native form. The GPCR is further linked to the cleavage site of the Tobacco Etch Virus nuclear-inclusion-a endopeptidase (TEV) and the tetracycline transactivator (tTA) transcription factor. The GPCR construct of choice is transfected into an engineered HEK293 cell line, HTLA, which contains a tTA-dependent luciferase gene and β-arrestin fused to the TEV endopeptidase. When a ligand binds the GPCR, the β-arrestin-TEV is recruited, resulting in cleavage of the tTA from the GPCR construct, thereby allowing it to enter the nucleus and initiate transcription of the luciferase reporter. Signal is finally detected by the addition of a luciferase substrate.^68^^,^^69^

The human PRESTO-Tango GPCR kit (AddGene #1000000068)^68^^,^^69^ was used in combination with the engineered HEK293 cell line HTLA. The HTLA cells were seeded at 75,000 cells/well in poly-D-lysine (Sigma, #P7886) coated white 96 well culture plates (Sigma #CLS3917) and incubated overnight. The cells were transfected with human Tango GPCR construct using a calcium transfection protocol as described for the IP3 assay, yet without using chloroquine. After incubating with DNA for 5 h, the medium was replaced with standard HTLA culture medium for incubation overnight. Next day, the cells were stimulated (see key resources table) with compounds dissolved in DMSO (Sigma #D5879), resulting in a final DMSO concentration of 1% for agonist mode and 2% for antagonist mode. For agonist stimulation, compounds were added for 18 h. For antagonist mode, the cells were pre-incubated with antagonist for 15 min, followed by addition of agonists for 18 h. Finally, luciferase activity was measured using steadylite plus Reporter Gene Assay System (PerkinElmer #6066759): The cells were washed in DPBS (Gibco, #14040–091), and 50 μL DPBS and 50 μL steadylite substrate was added to each well. The plates were incubated for 10 min at RT in the dark, and luminescence was measured on an Envision (PerkinElmer). All measurements were performed in duplicate. For agonist mode, the data was normalized to the baseline and maximum response of the reference agonist for each receptor. For antagonist mode, the data was normalized, so 100% reflected the response of the agonist in the absence of antagonist, and 0% was the baseline, i.e. the response in the absence of agonist and antagonist. EC50 values were calculated using nonlinear regression in GraphPad Prism.

#### RNA isolation

RNA was isolated from mouse organs using the RNeasy Lipid Tissue Mini kit (Qiagen #74804) and TissueLyser II (Qiagen) according to the manufacturer’s instructions, including the on-column DNAse digestion. The RNA was eluted with RNAse-free water and stored at −80°C. The RNA concentration and quality was measured on a NanoDrop ONE Spectrophotometer (Thermo Scientific).

RNA was purified from THP-1 cells, human neutrophils and murine neutrophils using the NucleoSpin RNA kit (Macherey Nagel #740955) according to the manufacturer’s instructions, including the on-column DNAse digestion. The RNA was eluted with RNAse free water and stored at −80°C. The RNA concentration and quality was measured on a NanoDrop ONE Spectrophotometer (Thermo Scientific).

#### cDNA synthesis

cDNA was synthesized from 500 ng mouse organ RNA, 100 ng human and murine neutrophil RNA or 250 ng THP-1 cell RNA using the Super-Script III kit (Thermo Fisher #18080044). RNA was mixed with sterile water (Amgros #744128) and 5x FS Buffer (Thermo Fisher #18080044), random primers at 3.2 μg/μL (Roche #11034731001), and 0.1 M dithiothreitol (Thermo Fisher #18080044). This was heated to 70°C for 3 min in an Eppendorf Vapo.protect Mastercycler® Pro (Eppendorf) and subsequently cooled on ice. Next, 10 mM dNTPs (Thermo Fisher #10297018), RNaseOUT (40 U/μL; Thermo Fisher #10777019) and Super-Script III (200U/μL; Thermo Fisher #18080044) were added, and the cDNA synthesis was run with the following program: 25°C for 5 min, 50°C for 60 min, 70°C for 15 min, followed by cooling to 4°C. A negative control sample with no RNA was prepared with all experiments. The cDNA was stored at −80°C.

#### qRT-PCR

Gene expression analyses were performed by qRT-PCR on a LightCycler® 480 II (Roche) using LigthCycler® 384 Multiwell Plates (Roche #04 724,749 001). PrecisionPLUS 2x qPCR MasterMix with SYBRgreen (Primer Design #JN190114-58256) was mixed with water (Amgros #744128) and primers to give a final primer concentration of 0.25 μM. Diluted cDNA was analyzed in triplicate for organs and neutrophils and single determinations for THP-1 cells. Murine *Ywhaz* or human *YWHAZ*^100^ was used as a reference gene. Threshold cycle (Ct) values ≥ 35 were discarded. The data was analyzed using the ΔΔCt method.^101^^,^^102^

#### Primer design

qRT-PCR primers were designed using the NCBI primer design tool (https://www.ncbi.nlm.nih.gov/tools/primer-blast/). PCR product size was set to 80–160 base pairs, and except for genes containing only one exon, the primers were designed so the amplicon would span across an intron. The specificity of each primer set was verified by BLAST. The primers were synthesized by Tag Copenhagen and diluted in sterile water (Amgros #744128). The specificity was verified by examination of melting curves showing only one peak across multiple cDNA dilutions. The efficiency was assessed by examining the slope of the linear increase in Ct values when plotting them against different cDNA dilutions.^101^^,^^103^ Primer sequences are found in Table S2.

#### Isolation of murine neutrophils

Murine neutrophils were isolated from the bone marrow of CONV-R male Swiss Webster mice of 9–14 weeks of age. Mice were euthanized by cervical dislocation, and the femurs and tibias were removed, sterilized in 70% ethanol (VWR #83801–360) and washed in PBS (SSC, UCPH). The bones were flushed with RPMI-1640 medium with NaHCO_3_ (SSC, UCPH) supplemented with 10% v/v FBS (Sigma, #F7524) and 2 mM EDTA (Sigma #03690–100 ML). The epiphyses were cut to small pieces and mixed with the bone marrow cells before dissociating cells by pipetting. The cells were passed through a 70 μm cell strainer (Falcon #352350) and centrifuged at 430x*g* for 7 min at 4°C. The cell pellet was subjected to hypotonic lysis with 0.2 and 1.6% NaCl solutions (Sigma #S7653-1KG) in MQ water, centrifuged as above, and resuspended in RPMI-1640 medium with NaHCO_3_ (SSC, UCPH) supplemented with 10% v/v FBS (Sigma #F7524) and 2 mM EDTA (Sigma #03690–100 ML). After another centrifugation, the cells were finally resuspended in cold PBS (SSC, UCPH), and neutrophils were isolated by density-gradient centrifugation using Histopaque-1119 (Sigma #11191–100 ML) and Histopaque-1077 (10771–100 ML). Finally, the interphase between Histopaque-1119 and -1077 was collected and washed twice in RPMI-1640 medium with NaHCO_3_ (SSC, UCPH) as above. Cell count and viability was measured using the NucleoCounter NC3000 (Chemometec). The cells were used for migration assays or qRT-PCR. For qRT-PCR, 1 × 10^6^ cells were pelleted and stored at −80°C until RNA isolation.

#### Isolation of murine monocytes

Murine monocytes were isolated from the bone marrow of CONV-R male Swiss Webster mice of 10–14 weeks of age. Mice were euthanized by cervical dislocation, and the femurs and tibias were removed, sterilized in 70% ethanol (VWR #83801–360) and washed in PBS (SSC, UCPH). The bones were flushed with MACS buffer (PBS (SSC, UPPH) supplemented with 0.5% BSA (Merck #12659) and 2 mM EDTA (Sigma #03690–100 ML)). The epiphyses were cut to small pieces and mixed with the bone marrow cells before dissociating cells by pipetting. The cells were passed through a 40 μm cell strainer (Falcon #352340). The cells were washed twice with MACS buffer, centrifuged at 300x*g* for 10 min at 4°C and resuspended in MACS buffer. Monocyte isolation was performed using the Monocyte Isolation Kit (BM) (Miltenyi Biotec #130-100-629), LS Columns (Miltenyi Biotec #130-042-401) and the QuadroMACS^TM^ Separator (Miltenyi Biotec #130-090-976), according to the manufacturer’s instructions. Cell count and viability was measured using the NucleoCounter NC3000 (Chemometec). The cells were used for migration assays.

#### Isolation of human peripheral neutrophils

Human peripheral blood neutrophils were isolated from healthy male volunteers of 20–40 years of age. Blood was drawn into VACUETTE® Blood Collecting Tubes coated with EDTA (Greiner bio-one #K3EDTA), and the majority of red blood cells were precipitated using 2% w/v dextran sulfate (Sigma #D8906) in a 0.9% NaCl solution (Mediq Danmark #B230553). Next, the upper phase was centrifuged for 10 min at 200x*g* at 4°C and the supernatant was discarded. The pellet was resuspended in 0.9% NaCl solution and neutrophils were separated from here using Lymphoprep (Axis-Shield #1114545) and density-gradient centrifugation, followed by a final hypotonic lysis of remaining red blood cells using sterile water (Mediq Danmark #B230534) followed by 1.8% NaCl (Sigma #S7653-1KG) in MQ water. Cell count and viability was measured using the NucleoCounter NC3000 (Chemometec). The cells were used for migration assays or qRT-PCR. For qRT-PCR, 1 × 10^6^ cells were pelleted and stored at −80°C until RNA isolation.

#### Neutrophil migration

Human peripheral neutrophils and murine bone marrow derived neutrophils were used in migration assays using the Boyden style Corning® HTS Transwell® 96 well system with 3 μm pores (Sigma #CLS3385). This assay system consists of a 96 well bottom plate as well as an insert for each well, containing a filter with pores for cells to migrate through. Compounds or vehicle (DMSO) in migration medium (RPMI-1640 with NaHCO_3_ (SSC, UCPH) with 0.5% w/v BSA (Sigma #A6003)) was added to the bottom well. The freshly isolated neutrophils (150,000 neutrophils in 75 μL migration medium) were added to the filter inserts. The plate was incubated for 1.5 h in a humidified atmosphere at 37°C and 5% CO_2_. Finally, the number of cells that had migrated to the bottom chamber was assessed using the CellTiter-Glo® reagent (Promega #G7570) according to the manufacturer’s instructions. Luminescence was measured on an Envision (PerkinElmer). In experiments where the effect of PTX was investigated, the freshly isolated human neutrophils were pre-stimulated with PTX (Sigma #P2980) (200 ng/mL) in migration medium for 2 h in a humidified atmosphere at 37°C and 5% CO_2_, washed twice in RPMI-1640 medium with NaHCO_3_ (SSC, UCPH) and then used in the migration assay as described above. All analyses were performed in duplicate. Migration index (MI) was calculated as the relative migration of samples compared to the vehicle control. The DMSO concentration of all conditions was 0.1%.

#### THP-1 and monocyte migration

The THP-1 cells and primary murine monocytes were used in migration assays using the Boyden style Corning® HTS Transwell® 96 well system with 5 μm pores (Sigma #CLS3388). This assay system consists of a 96 well bottom plate as well as an insert for each well, containing a filter with pores for cells to migrate through. Compounds or vehicle (DMSO) in migration medium (RPMI-1640 with NaHCO_3_ (SSC, UCPH) with 0.5% w/v BSA (Sigma #A6003)) was added to the bottom well. The THP-1 cells (100,000 neutrophils in 75 μL migration medium) were added to the filter inserts. The plate was incubated for 3 h in a humidified atmosphere at 37°C and 5% CO_2_. Finally, the number of cells that had migrated to the bottom chamber was assessed using the CellTiter-Glo® reagent (Promega #G7570) according to the manufacturer’s instructions. Luminescence was measured on an Envision (PerkinElmer). All analyses were performed in duplicate. Migration index (MI) was calculated as the relative migration of samples compared to the vehicle control. The DMSO concentration of all conditions was 0.1%.

### Quantification and statistical analysis

#### Statistical analyses

Unless otherwise stated in the figure or table legends, data is shown as means ± SEM The N for each experiment is found in the figure legends, where N represents the number of animals used for animal experiments; or the number of experimental replicates for *in vitro* assays. For the clinical baseline data, Fisher's Exact test was used for dichotomous variables and Student’s *t* test or Wilcoxon rank-sum test were used as appropriate for continuous variables. One-way ANOVA with Tukey’s multiple comparison test was used to compare the effect of signaling inhibitors on 3-hydroxydecanoate signaling in the xCELLigence assay. One-way ANOVA with Dunnett’s multiple comparison test was used when comparing migration of MCFAs to the DMSO control. The fasting-refeeding experiment was analyzed using the repeated measures Friedman’s test with Dunn’s post-hoc analysis. Analysis of effects of 3-hydroxydecanoate on mouse physiology (ipGTT, body weight, fat mass, lean mass) was done using a two-way ANOVA with repeated measurements and Sidak’s multiple comparison test. The same test was used in migration experiments where neutrophils were pre-incubated with or without PTX. Otherwise, two-tailed Mann-Whitney test was used when only two groups were compared, as indicated. Significance was defined as p < 0.05.

## Data Availability

This study did not generate new code. Data reported in this paper will be shared by the lead contact upon request.
