# Supplementary material for: Type 2 diabetes is associated with increased circulating levels of 3-hydroxydecanoate activating GPR84 and neutrophil migration
Source: iScience. 2022 Nov 26;25(12):105683. doi: 10.1016/j.isci.2022.105683 (PMC9763857; doi:10.1016/j.isci.2022.105683)
Supplement: Document S1. Figures S1–S6, Tables S1 and S2 [file mmc1.pdf]

iScience, Volume *25*

Supplemental information

Type 2 diabetes is associated with increased circulating levels of 3-hydroxydecanoate activating GPR84 and neutrophil migration

Randi Bonke Mikkelsen, Tulika Arora, Kajetan Trošt, Oksana Dmytriyeva, Sune Kjærsgaard Jensen, Abraham Stijn Meijnikman, Louise Elisabeth Olofsson, Dimitra Lappa, Ömrüm Aydin, Jens Nielsen, Victor Gerdes, Thomas Moritz, Arnold van de Laar, Maurits de Brauw, Max Nieuwdorp, Siv Annegrethe Hjorth, Thue Walter Schwartz, and Fredrik Bäckhed

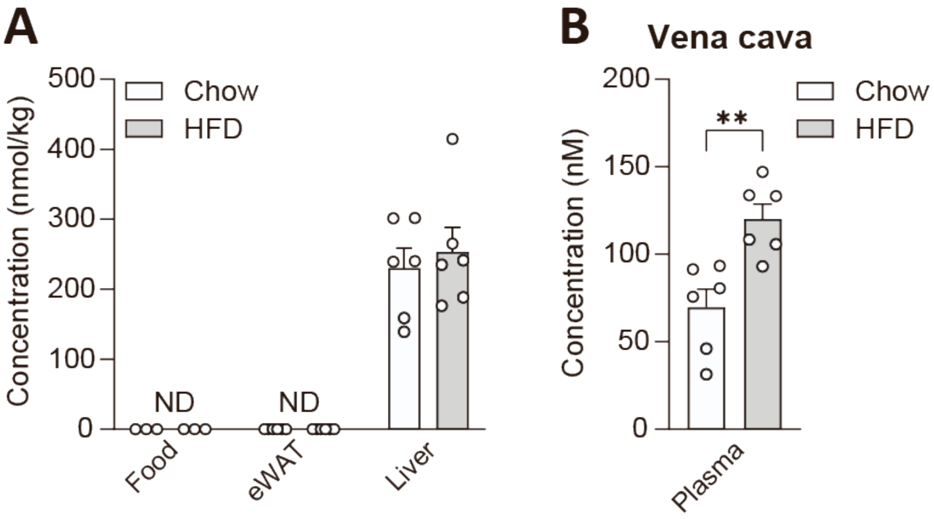

Figure S1: 3-hydroxydecanoate levels in food, tissues and plasma. Related to Figure 1. A) Levels of 3-hydroxydecanoate (3-OH-C10) in chow and HFD (N = 3), and eWAT and liver from CONV-R mice fed chow or HFD (N = 6). ND = Not detected. **B)** Plasma levels of 3-hydroxydecanoate in vena cava from CONV-R mice fed chow or HFD (N = 6). Mice were fasted for 4 hours before sampling. Data is shown as mean ± SEM. ** $P < 0.01$. *P* values were determined by two-tailed Mann-Whitney test.

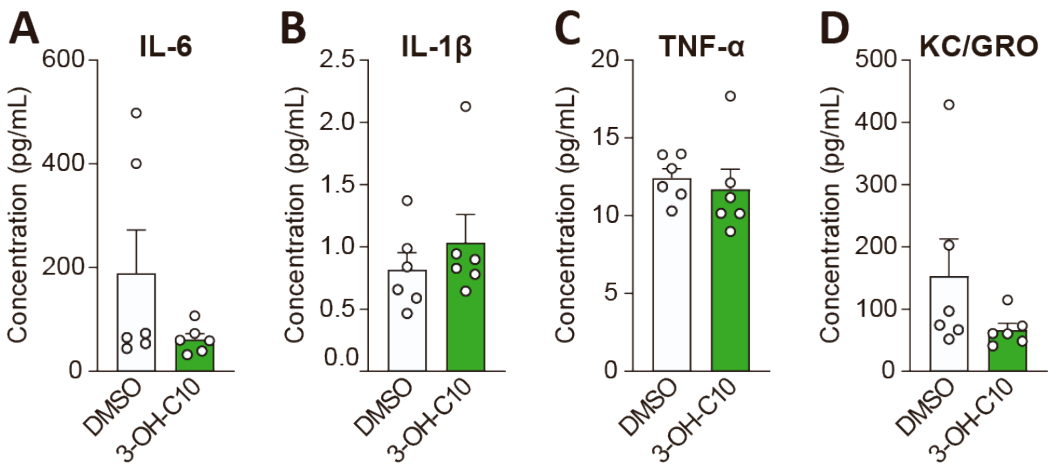

Figure S2: 3-hydroxydecanoate does not increase plasma cytokine levels. Related to Figure 3. A-D) Murine vena cava plasma levels of A) IL-6, B) IL-1β, C) TNF-α and D) KC/GRO after dosing DMSO vehicle or 3-hydroxydecanoate (3-OH-C10) for seven days. Data is shown as mean ± SEM. *P* values were determined by two-tailed Mann-Whitney U test.

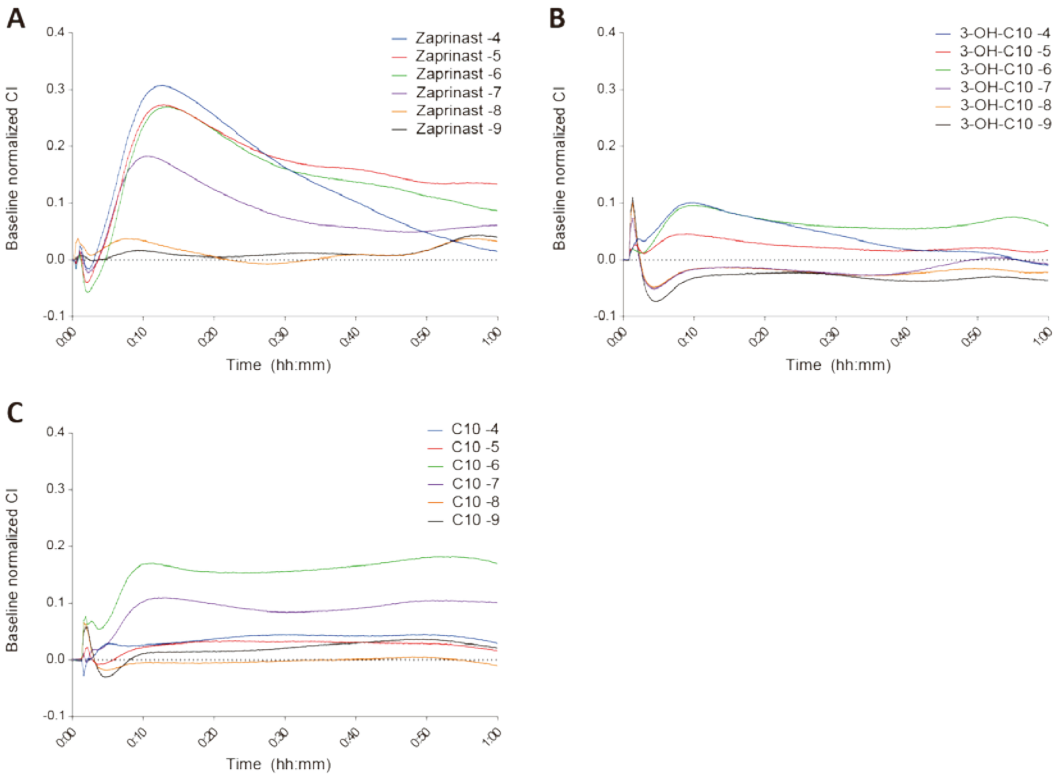

Figure S3: Representative baseline normalized CI traces of HT29 cells stimulated in the xCELLigence assay. Related to Figure 4. A-C) Representative example of stimulation of HT29 cells with A) zaprinast, B) 3-hydroxydecanoate (3-OH-C10) and C) decanoate (C10).

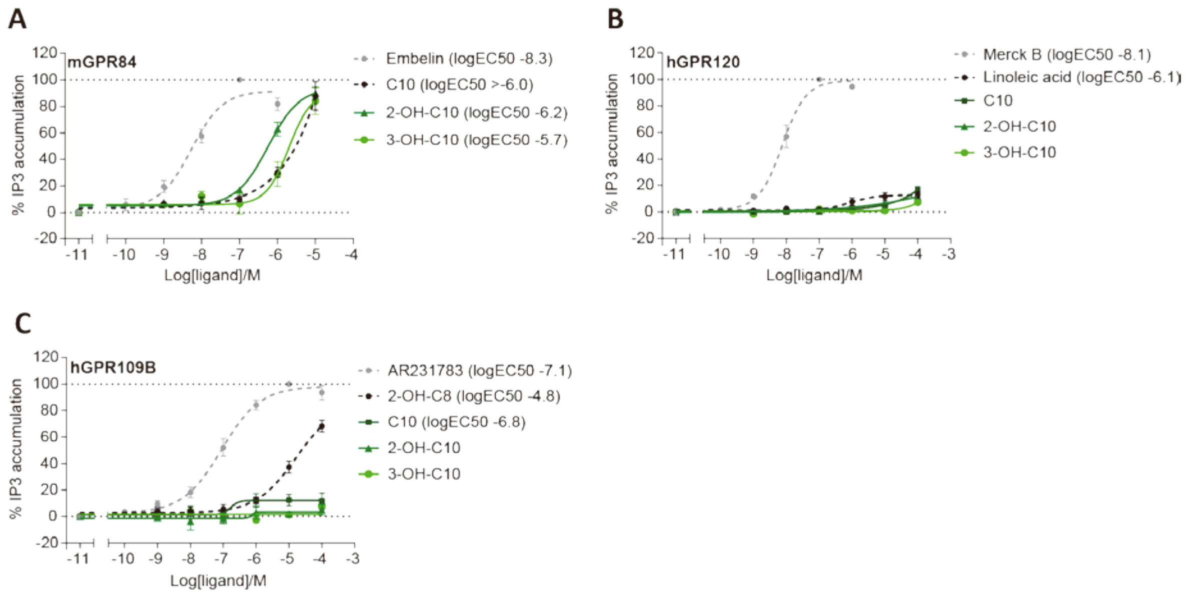

Figure S4: 3-hydroxydecanoate is an agonist for murine GPR84, but not human GPR120 or GPR109B in an IP3 accumulation assay. Related to Figure 5. A) IP3 accumulation assay of COS-7 cells transfected with mouse GPR84 and Gα$_{\Delta 6qi4myr}$ and stimulated as indicated (N = 3). **B)** IP3 accumulation assay of COS-7 cells transfected with human GPR120 and stimulated as indicated, including the endogenous GPR120 agonist linoleic acid and the synthetic reference agonist Merck B (Example 209 in[1]) (N = 3). **C)** IP3 accumulation assay of COS-7 cells transfected with human GPR109B and Gα$_{\Delta 6qi4myr}$ and stimulated as indicated, including the endogenous agonist 2-hydroxyoctanoate (2-OH-C18) and the synthetic reference agonist AR231783 (N = 3). Data is shown as normalized mean ± SEM.

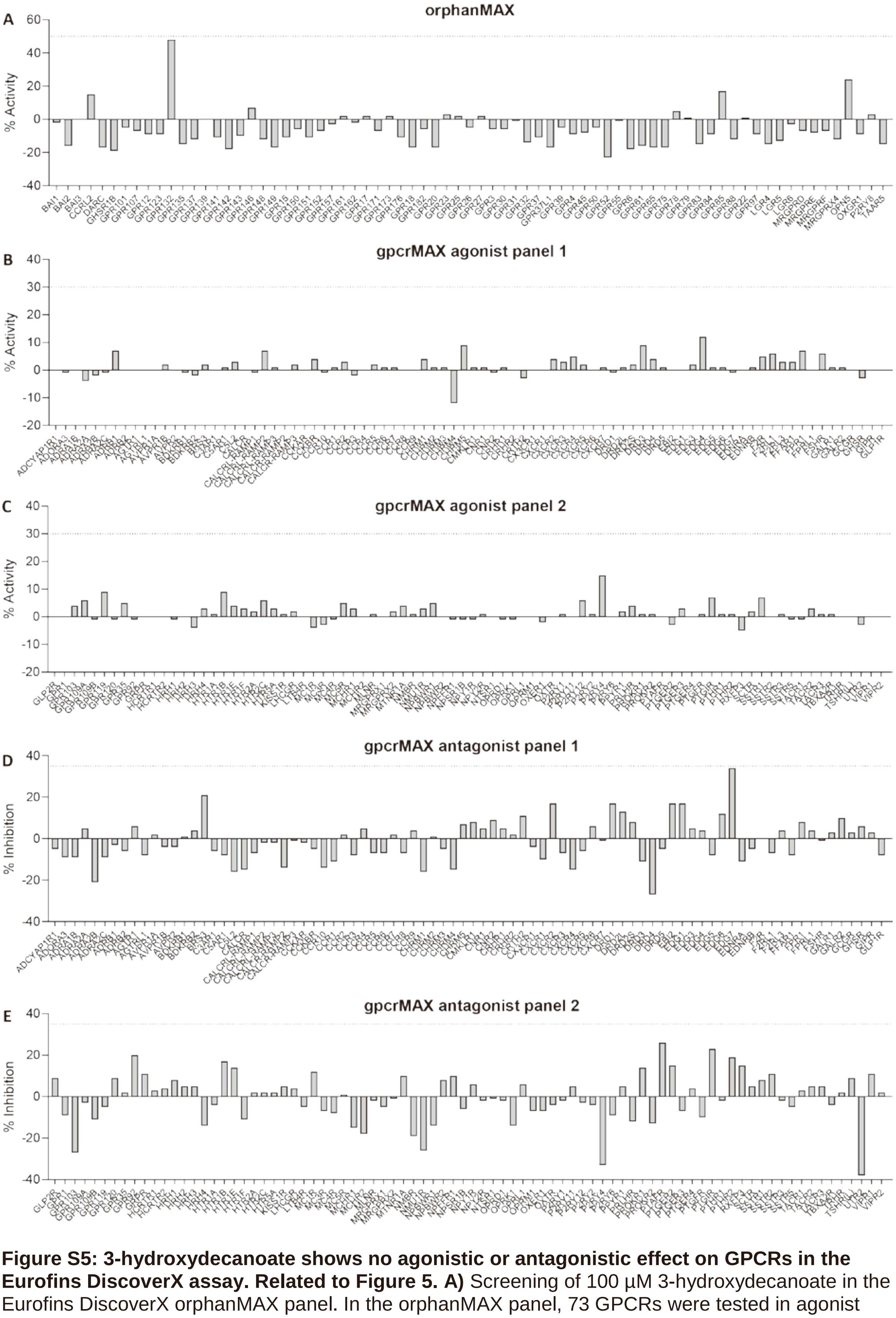

Figure S5: 3-hydroxydecanoate shows no agonistic or antagonistic effect on GPCRs in the Eurofins DiscoverX assay. Related to Figure 5. A) Screening of 100 µM 3-hydroxydecanoate in the Eurofins DiscoverX orphanMAX panel. In the orphanMAX panel, 73 GPCRs were tested in agonist mode. According to the provided guidelines, the compound was considered an agonist, if it increased activity by >50% in the orphanMAX, as indicated by the horizontal line at 50% activity (N = 1). **B-C)**

Screening of 100 µM 3-hydroxydecanoate in the Eurofins DiscoverX gpcrMAX panel in agonist mode. Here, 168 GPCRs were tested. According to the provided guidelines, the compound was considered an agonist, if it increased activity by >30% in the gpcrMAX, as indicated by the horizontal line at 30% activity (N = 1). **D-E)** Screening of 100 µM 3-hydroxydecanoate in the Eurofins DiscoverX gpcrMAX panel in antagonist mode. Here, 168 GPCRs were tested. According to the provided guidelines, the compound was considered active, if it inhibited activity by >35% in the gpcrMAX, as indicated by the horizontal line at 35% inhibition (N = 1).

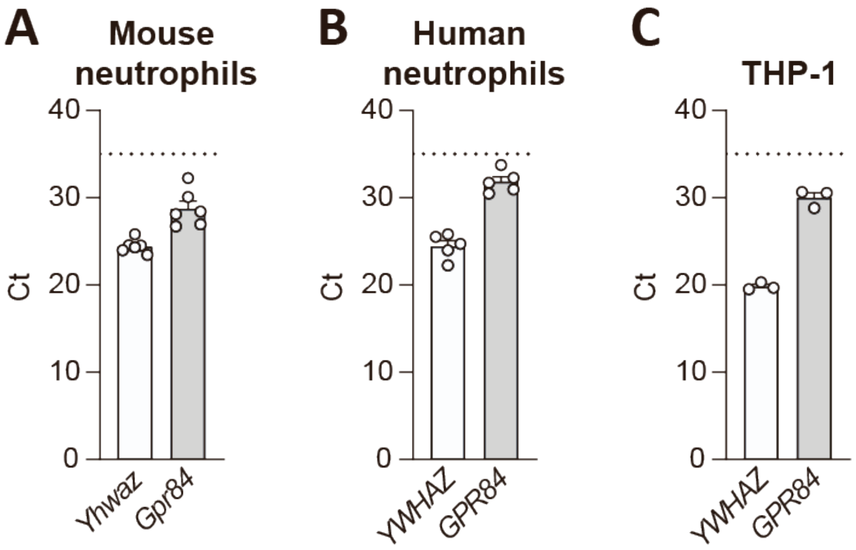

Figure S6. Related to Figure 6 and 3: Expression of the human and murine GPR84 gene in immune cells. A) Gene expression of mouse *Gpr84* in murine bone marrow-derived neutrophils. **B-C)** Gene expression of human *GPR84* in B) human primary neutrophils and C) the human monocyte cell line THP-1. Data is shown as mean ± SEM.

Table S1: LogEC50 values for the *in vitro* IP3 accumulation assays. Related to Figure 5.

**Compound**		**Transfected GPCR**	**Main endogenous G protein[2]**	**Transfected $G\alpha_{\Delta 6qi4myr}$**	**logEC50 (efficacy)**	**EC50**
Embelin		GPR84	$G\alpha_i$ + more	Yes	-7.9 (100%)	12.6 nM
C4	Butyrate	GPR84	$G\alpha_i$ + more	Yes	0	0
C6	Hexanoate	GPR84	$G\alpha_i$ + more	Yes	-5.6 (23%)	2.51 µM
C8	Octanoate	GPR84	$G\alpha_i$ + more	Yes	-4.0 (65%)	100 µM
2-OH-C8	2-hydroxyoctanoate	GPR84	$G\alpha_i$ + more	Yes	0	0
3-OH-C8	3-hydroxyoctanoate	GPR84	$G\alpha_i$ + more	Yes	0	0
C10	Decanoate	GPR84	$G\alpha_i$ + more	Yes	-5.6 (73%)	2.51 µM
C10	Decanoate	GPR40 (FFA1)	$G\alpha_q$	No	>-4	0
C10	Decanoate	GPR120 (FFA4)	$G\alpha_i/G\alpha_q$	No	0	0
C10	Decanoate	GPR109B (HCA3)	$G\alpha_i$	Yes	-6.8 (12%)	158.5 nM
2-OH-C10	2-hydroxydecanoate	GPR84	$G\alpha_i$ + more	Yes	-6.0 (84%)	1 µM
2-OH-C10	2-hydroxydecanoate	GPR40 (FFA1)	$G\alpha_q$	No	0	0
2-OH-C10	2-hydroxydecanoate	GPR120 (FFA4)	$G\alpha_i/G\alpha_q$	No	0	0
2-OH-C10	2-hydroxydecanoate	GPR109B (HCA3)	$G\alpha_i$	Yes	0	0
3-OH-C10	3-hydroxydecanoate	GPR84	$G\alpha_i$ + more	Yes	-5.5 (74%)	3.16 µM
3-OH-C10	3-hydroxydecanoate	GPR40 (FFA1)	$G\alpha_q$	No	0	0
3-OH-C10	3-hydroxydecanoate	GPR120 (FFA4)	$G\alpha_i/G\alpha_q$	No	0	0
3-OH-C10	3-hydroxydecanoate	GPR109B (HCA3)	$G\alpha_i$	Yes	0	0
3-OH-C10	3-hydroxydecanoate	GPR43 (FFA2)	$G\alpha_i/G\alpha_q$	No	0	0
3-OH-C10	3-hydroxydecanoate	GPR41 (FFA3)	$G\alpha_i$	Yes	0	0
3-OH-C10	3-hydroxydecanoate	GPR81 (HCA1)	$G\alpha_i$	Yes	0	0
3-OH-C10	3-hydroxydecanoate	GPR109A (HCA2)	$G\alpha_i$	Yes	0	0
3-OH-C10	3-hydroxydecanoate	GPR35	Gi/G13	Yes	0	0
3-OH-C10	3-hydroxydecanoate	GPR91	$G\alpha_i/G\alpha_q$	Yes	0	0
3-OH-C10	3-hydroxydecanoate	GPR142	$G\alpha_i/G\alpha_q$	Yes	0	0
3-OH-C10	3-hydroxydecanoate	FPR1	$G\alpha_i/G\alpha_q$	Yes	0	0
C12	Dodecanoate, laurate	GPR84	$G\alpha_i$ + more	Yes	>-5	>10 µM
3-OH-C12	2-hydroxylaurate	GPR84	$G\alpha_i$ + more	Yes	-6.0 (90%)	1 µM
C14	Tetradecanoate, myristate	GPR84	$G\alpha_i$ + more	Yes	-4.6 (34%)	25.1 µM
2-OH-C14	2-hydroxymyristate	GPR84	$G\alpha_i$ + more	Yes	-6.1 (73%)	794.3 nM
3-OH-C14	3-hydroxymyristate	GPR84	$G\alpha_i$ + more	Yes	>-6	>1 µM
di-C10	Decanedioate, sebacate	GPR84	$G\alpha_i$ + more	Yes	0	0
3-OH-diC10	3-hydroxysebacate	GPR84	$G\alpha_i$ + more	Yes	0	0

The tested compounds are indicated with their chemical names and abbreviations used in figures. The transfected GPCR cDNA is indicated as well as whether the cells were co-transfected also with the chimeric G protein. A "0" indicates that no effect was observed from a particular compound on the indicated GPCR. The logEC50 values were calculated in GraphPad Prism using non-linear regression. The percentages indicate efficacy compared to that of the relevant reference agonist where a response of the compound was observed (embelin for GPR84, TAK-875 for GPR40, Merck B for GPR120 and AR231783 for GPR109B). For unsaturated curves, no efficacy is indicated, and the potencies are indicated as being above a certain concentration.

Table S2: qRT-PCR primer sequences. Related to STAR Methods.

**Gene**	**Species**	**Forward (5'-3')**	**Reverse (5'-3')**	**Amplicon size, bp**	**Ref. no.**
Ywhaz	Mouse	AGACGGAAGGTGCTGAGAAA	GAAGCATTGGGGATCAAGAA	127	NM_001253805.1
Tnf	Mouse	GTAGCCCACGTCGTAGCAAA	TTGAGATCCATGCCGTTGGC	95	NM_013693.3
Il1b	Mouse	GCCACCTTTTGACAGTGATGAG	GACAGCCCAGGTCAAAGGTT	95	NM_008361.4
Ccl2	Mouse	GCCTGCTGTTCACAGTTGC	TCTCCAGCCTACTCATTGGGA	123	NM_011333.3
Tgfb	Mouse	ATGCTAAAGAGGTCACCCGC	TGCTTCCCGAATGTCTGACG	119	NM_011577.2
Cxcl1	Mouse	ACTCAAGAATGGTCGCGAGG	GTGCCATCAGAGCAGTCTGT	123	NM_008176.3
Il6	Mouse	AGTCCTTCCTACCCCAATTTCC	TGGTCTTGGTCCTTAGCCAC	80	NM_031168.2
Itgax	Mouse	GGCTGCAAGCATCATTCGTT	GCATCAAAGTTCTCCACGCT	132	NM_021334.3
Ly6g	Mouse	CTTGTCACTGTGCCTGCAAC	AATTGTAGCACTCCAGCCCC	157	NM_001310438.1
Gpr84	Mouse	AGGTGACCCGTATGTGCTTC	CAGCCACCATGTGCACTACT	120	NM_030720.2
YWHAZ	Human	TGAAGCCATTGCTGAACTTG	CTCCTGCTTCAGCTTCGTCT	131	NM_003406
GPR84	Human	CATGAGTCTGTGCTGGGCTA	GCCAGTGTGAGGTTGGCTAT	158	NM_020370.2

Reference List

1. Shi, D.F., Song, J.S., Ma, J., Novack, A., Pham, P., Nashashibi, I., Rabbat, C.J., and Chen, X. (2010) GPR120 receptor agonists and uses thereof. patent application PCT/US2009/068576.
2. Husted, A.S., Trauelsen, M., Rudenko, O., Hjorth, S.A., and Schwartz, T.W. (2017). GPCR-Mediated Signaling of Metabolites. Cell Metab *25*, 777-796.
